# Biobjective gradient descent for feature selection on high dimension, low sample size data

**DOI:** 10.1371/journal.pone.0305654

**Published:** 2024-07-18

**Authors:** Tina Issa, Eric Angel, Farida Zehraoui

**Affiliations:** Universite Paris-Saclay, Univ Evry, IBISC, Evry-Courcouronnes, France; New York University Abu Dhabi, UNITED ARAB EMIRATES

## Abstract

Even though deep learning shows impressive results in several applications, its use on problems with High Dimensions and Low Sample Size, such as diagnosing rare diseases, leads to overfitting. One solution often proposed is feature selection. In deep learning, along with feature selection, network sparsification is also used to improve the results when dealing with high dimensions low sample size data. However, most of the time, they are tackled as separate problems. This paper proposes a new approach that integrates feature selection, based on sparsification, into the training process of a deep neural network. This approach uses a constrained biobjective gradient descent method. It provides a set of Pareto optimal neural networks that make a trade-off between network sparsity and model accuracy. Results on both artificial and real datasets show that using a constrained biobjective gradient descent increases the network sparsity without degrading the classification performances. With the proposed approach, on an artificial dataset, the feature selection score reached 0.97 with a sparsity score of 0.92 with an accuracy of 0.9. For the same accuracy, none of the other methods reached a feature score above 0.20 and sparsity score of 0.35. Finally, statistical tests validate the results obtained on all datasets.

## Introduction

Deep learning has gained popularity in various fields, especially when a large amount of data is accessible. However, in some research fields, the available data falls in the High Dimensions, Low Sample Size category (HDLSS). For example, in bioinformatics, predicting phenotypes from genetic variants is challenging due to high dimensionality and small number of patients. Similar problems are found in other areas, such as chemistry, financial engineering, and computer vision. When dealing with HDLSS data, most machine learning algorithms face severe overfitting and high-variance gradients [1]. Feature selection is among the solutions proposed to overcome these problems. Feature selection in deep learning can be considered as a particular type of architecture pruning, where neurons are eliminated from the input layer rather than the hidden layers [2]. This approach is a form of sparsity, where unneeded weights are set to zero during training. Recent research has focused on promoting sparsity in deep neural networks to improve their efficiency. Sparsity-based feature selection has proven to be effective in handling HDLSS data. Various techniques have been proposed, including dropout-based methods [3, 4] and methods that add a regularization term to the network’s loss function [5, 6].

Either limited sparsity or regularization term in the objective function is used to promote global network sparsity. In addition to feature selection, sparsity should be sought to reduce a network’s complexity and running time. Methods based on regularization use a single scalarized objective function consisting of a loss function that computes a network’s error and a regularizer that reduces the network’s weights and increases its sparsity. This approach has several disadvantages. First, the regularizer is controlled by a hyperparameter whose value may be difficult to set. Second, for a fixed hyperparameter, minimizing such a scalarized objective function gives only one solution. However, when dealing with conflicting objectives, such as loss and sparsity, it is generally not possible to find one optimal solution for both objectives at the same time, and one looks instead for trade-off solutions, the so-called Pareto set. Lastly, by varying the hyperparameter value, these methods can get points from the Pareto set only when the Pareto front is convex [7]. Sener and Koltun [8] proposed a multiobjective approach for multitask learning to get a single Pareto optimal solution with a good trade-off among different tasks. Based on this work, Lin et al. [9] developed a new Pareto multitask learning algorithm (ParetoMTL) to get a set of well-distributed Pareto optimal solutions. While using multiobjective optimization to perform feature selection can improve the classification results, it has been limited to evolutionary and metaheuristics algorithms [10, 11]. These algorithms were not tested on HDLSS data and do not include the feature selection process into the learning phase. Additionally, most proposed algorithms, whether based on single objective optimization or multiobjective, focus on reducing the number of features without looking at which features were selected. This can be seen as a disadvantage as explainable models are increasingly demanded. To overcome this gap, this paper proposes a new Biobjective Feature Selection method (BFS) designed for HDLSS data that performs feature selection based on network sparsification. The approach is based on a constrained bicriteria gradient descent inspired from [9] with some improvements. The proposed method takes into account the large difference of scales on the two objective functions and improves the computational complexity of the algorithm.

The method was tested on HDLSS classification problems on both artificial and real datasets. It was shown that the method can perform better in terms of feature selection and sparsity while maintaining a high accuracy. The main contributions of this work are:

- Proposing a new approach feature selection based on network sparsification for HDLSS data based on biobjective gradient descent.
- Developing a novel formulation of the constrained biobjective optimization problem with fewer constraints compared to the literature. By doing so, this method can be applied regardless of the number of regions in the objective space.
- Introducing a normalization step of the objective functions to ensure an objective function homogeneity requirement.
- Evaluating the method on HDLSS classification problems, both artificial and real.

This article is organized as follows. First, a summary of current works found in the literature is given. Then, details about the proposed method are presented followed by the obtained results and their discussion. Lastly, a conclusion, including a few perspectives and some limitations of the current approach, is presented.

## Related works

As mentioned before, there are different feature selection approaches. Some of these approaches are based on neural networks. A summary of some state-of-the-art methods is given in this section. The focus is on methods embedding Feature Selection (FS) in the learning step of the algorithm. In addition, since this work is based on a biobjective gradient descent optimization algorithm, some popular multiobjective based approaches used in the field of neural networks are mentioned.

### Feature selection

While most feature selection methods based on deep neural networks require a large number of training data and fail on HDLSS data, there exist few methods specifically tailored to overcome this problem. Deep Neural Pursuit (DNP) [4] is an MLP that uses multiple dropouts to learn important features. GRACES [12] is a method inspired by DNP. Still, it is based on graph convolution networks and uses latent relations between the samples in addition to multiple dropouts to improve feature selection. In [13], the authors propose DeepFS, a method combining autoencoders and feature extraction to select the relevant features. Other methods are based on stochastic gates. For example, in [14], the authors add stochastic gates to the input layer to perform feature selection: an open gate means the feature is important. Similarly, in [15], the authors use stochastic gates to implement what they call a locally sparse network. Their method can detect the important features that are specific to each sample. In [6], the authors add a FS layer that satisfies two constraints in order to select the relevant features. However, while this method gives a sparse input layer with only relevant features, it only works with a specific MLP architecture.

Other methods use regularization to perform feature selection based on sparsity.

The most popular example of regularization is the lasso (*l*_1_) [16]. Other variants include sparse group lasso [5], and using both *l*_1_ and *l*_1/2_ [17]. In DNN, applying *l*_1_ regularization (lasso) consists of shrinking the network’s connections weights’ towards zero. However, as each neuron has many connections, the probability of removing a neuron from a network is very low. Therefore, new methods extending the lasso and considering this problem emerged. For instance, group lasso [18], defined as the (2,1)-norm, consists of shrinking to 0 a group of connections simultaneously rather than one at a time. The group of connections is usually made of all outgoing connections of one neuron, and eliminating it means canceling the neuron. Sparse group lasso [19] was proposed as a method to take advantage of the sparsity induced by both group lasso and lasso.

The regularization can be applied only to the input layer or to the whole network. When applied to the input layer, a lasso [16] or a group lasso [18] regularizer shows relevant feature selection results. When applied to the whole network, in addition to input feature selection, high-level feature selection is performed. This can lead to better results when dealing with HDLSS problems and can improve network interpretation.

In [5], the authors show how using group lasso and specifically sparse group lasso is effective for creating sparse networks. Yoon and Hwang [20] combine this group sparsity with an exclusive sparsity based on (1,2)-norm to introduce diversity into the selected weights.

### Multiobjective optimization

One aim of multiobjective optimization is to find the Pareto set that is constituted of Pareto optimal solutions, i.e. non-dominated solutions (see S1 Appendix for definitions). Pareto optimization approaches became more widely used in the field of machine learning in general and deep learning specifically [21] to overcome the disadvantages of the scalarization approach. There exist different types of algorithms to solve multiobjective optimization problems. One can cite methods based on Evolutionary Algorithms (EA) and methods based on gradient descent. Evolutionary Algorithms (EA) combining genetic algorithms and neural networks [22, 23] are the most popular approach, and many variants have been proposed in recent works [24–27]. However, because of the nature of these algorithms, learning relevant features is not done during the training step of the classifier. In addition, EA methods have been shown to be time-consuming for FS tasks [24].

As an alternative, metaheuristics based algorithms, are therefore a popular choice for FS problems. In [28], the authors introduced a FS method that uses a Particle Swarm Optimization (PSO) and a modified Firefly Algorithm (FA) inspired by quantum computing to find optimal Convolutional Neural Networks (CNNs). Optimization of their neural networks is based on two objective functions, the model’s accuracy and the ratio between the number of selected features by the model and the overall number of features. In [29], the authors propose to combine Brain Storm Optimization, a metaheuristics algorithm to FA in order to select features. They use K-Nearest Neighbors, a supervised learning algorithm, for the classification task. In [11], the authors opted for a modified version of the FA to propose a hybrid two-level framework to achieve FS and hyperprameter tuning of an eXtreme Gradient Boosting (XGBoost) machine learning model. Similarly, in [10], the authors propose a two-level framework based on a novel diversity oriented social network search metaheuristics algorithm. The algorithm is used to achieve FS and machine learning tuning. However, in both methods, the FS is done in the first level and is fed as an input to the second level of the framework. In other works, a metaheuristic algorithm is combined with a Genetic Algorithm (GA). For instance, in [30], the authors combine Assimilated Artificial Fish Swarm Optimization (AAFSO) and GA with a CNN. AAFSO is used for feature selection and the GA for choosing the hyperparameters of the CNN. In [31], the authors use a qualitative approximation approach to adapt both GA and PSO to get models that converge faster. This makes it possible to test such algorithms on large datasets. Wu et al. [32] introduced a federated deep fuzzy neural network that uses PSO to optimize the network’s architecture based on four objective functions for Epistatic Detection. The fuzzy logic makes it easier to interpret the neural network’s results and thus to detect the important genes causing a disease. However, these methods, just like EA methods, require the feature selection step to be evaluated separately from the training step. In addition, none of these methods have been specifically designed for HDLSS data.

Gradient-based methods can therefore be more interesting for FS tasks. In practice, finding Pareto optimal solutions is difficult, and several works define necessary conditions for Pareto optimality. The most used are Pareto criticality [33] and Pareto stationarity [34]. Fliege and Svaiter [33] proposed a generalization of the single objective steepest descent algorithm based on Pareto criticality, while Desideri [34] proposed another approach based on Pareto stationarity (See S1 Appendix for more details). While reported in the literature, the equivalence between Pareto stationarity and Pareto criticality definitions was never formally proven before, and sometimes the terms are used interchangeably [35]. Another observation that was reported in the literature is the strong link between the two optimization problems defined by Fliege and Svaiter and Desideri via the duality theory [33]. However, no detailed proof was provided. A formal proof of the equivalence between the two definitions is presented in S1 Appendix. Furthermore, a formal proof demonstrating the equality of the two directions computed by the Desideri approach and the Fliege and Svaiter approach, is provided in S1 Appendix.

In recent years, multiobjective gradient descent algorithms have been increasingly used in DNNs [36]. So far, these algorithms have been mostly used for multi-task learning [8, 9, 37] and not for FS for HDLSS data. In [8], the authors aim to obtain one solution with a trade-off for both tasks. In [9], Lin et al. developed ParetoMTL, a constrained multiobjective gradient descent-based method, to generate multiple networks with different trade-offs between the tasks. However, ParetoMTL has some limitations, particularly in its inability to generate dispersed networks when tasks have varying levels of difficulty. In [37] the authors developed multiple algorithms to obtain Exact Pareto Solution (EPO) for multi task problems and multi-criteria decision making. Others base their work on hypernetworks first introduced by [38] in order to find a continuous Pareto front. In [39], the authors use multi-sample hypernetworks and hypervolume indicator maximization for multi task learning. However, none of these methods have been tested for feature selection purposes.

## Materials and methods

### General notations

Given a training set of *N* inputs *X* = {*X*_1_, …, *X*_*N*_}, ($X_{i}\in R^{p},1\leq i\leq N$), with *p* the number of features such as *p* > >*n*, and their corresponding labels *Y* = {*Y*_1_, …, *Y*_*N*_} over *K* classes {*c*_1_, …, *c*_*K*_}, the goal is to predict the example classes. *Y*_*i*_, for 1 ≤ *i* ≤ *N*, is represented as a one-hot encoded vector of dimension *K*, i.e. *Y*_*i*_ = (*Y*_*i*1_, …, *Y*_*iK*_) with *Y*_*ik*_ = 1 if *Y*_*i*_ corresponds to class *c*_*k*_ and *Y*_*ik*_ = 0 otherwise.

Let *L* be the layers of a neural network, with *l* = 0 (resp. *l* = *L*) the input (resp. output) layer. For $h_{j}^{l}$, the *j*^*th*^ neuron of the layer *l* (1 ≤ *l* ≤ *L*), its activation value $a_{j}^{l}$ is defined by $a_{j}^{l}=\phi \left(z_{j}^{l}\right)$ where:

$$\begin{array}{c} z_{j}^{l}=\sum_{i=1}^{H_{l-1}}w_{ij}^{l}a_{i}^{l-1}+b_{j}^{l}, \end{array}$$

with *H*_*l*_ the size of the layer *l*, $w_{ij}^{l}$ the weight of the connection from neuron $h_{i}^{l-1}$ to neuron $h_{j}^{l}$, $b_{j}^{l}$ the bias of $h_{j}^{l}$, *ϕ* its activation function and $a_{i}^{0}$ the *i*^*th*^ input feature. For the hidden layers (1 ≤ *l* ≤ *L* − 1), the used activation function is the rectified linear unit function (ReLU) defined as *ϕ*(*z*) = max(0, *z*). As for the output layer *L*, the used activation function is the softmax function $\phi :R^{K}\to {\left(0,1\right)}^{K}$ defined as:

$$\begin{array}{c} \phi {\left(z^{L}\right)}_{k}=\frac{exp(z_{k}^{L})}{\sum_{i=1}^{K}exp\left(z_{i}^{L}\right)}, \end{array}$$

with 1 ≤ *k* ≤ *K* and $z^{L}=\left(z_{1}^{L},\ldots ,z_{K}^{L}\right)$. The values *ϕ*(*z*^*L*^)_*k*_ represent the posterior probabilities, estimated by the network, that a given input belongs to a class *c*_*k*_, for 1 ≤ *k* ≤ *K*.

The neural network is represented by its set of parameters $\theta =\left\{w_{ij}^{l};l=1,\ldots ,L;i=1,\ldots ,H_{l-1};j=1,\ldots ,H_{l}\right\}\cup \left\{b_{i}^{l};l=1,\ldots ,L;i=1,\ldots ,H_{l}\right\}$. $\theta_{i}^{l}=\left(w_{i}^{l},b_{i}^{l}\right)$ is the vector representing the weights $w_{i}^{l}=\left(w_{1i}^{l},\ldots ,w_{H_{l-1}i}^{l}\right)$ and the bias $b_{i}^{l}$ of the neuron $h_{i}^{l}$.

For an input *X*_*i*_, the output of the neural network is the vector:

$$\begin{array}{c} \tilde{Y_{i}}\left(X_{i},\theta \right)=\left(a_{1}^{L},\ldots ,a_{K}^{L}\right), \end{array}$$

where $a_{k}^{L}=\phi {\left(z^{L}\right)}_{k}$ for 1 ≤ *k* ≤ *K*. ${\tilde{Y}}_{ik}\left(X_{i},\theta \right)$ is the *k*-th component of the vector $\tilde{Y_{i}}\left(X_{i},\theta \right)$, i.e. ${\tilde{Y}}_{ik}\left(X_{i},\theta \right)=a_{k}^{L}$.

### Choice of the objective functions

As stated before, feature selection is performed through network sparsification. Suppose all the outgoing connections from an input neuron are close to zero, whereas the accuracy of the model is still high. In that case, this input feature is irrelevant to the classification task. Moreover, since the sparsity of the networks is also a desirable property, sparsity is enforced beyond the input layer, i.e., also in the hidden layers. Beyond a certain level of sparsity, the accuracy will decrease. So, the goal of this method is to balance the network’s accuracy and its sparsity, which is achieved through a biobjective optimization approach. In the sequel, experiments are run on Multi-Layer Perceptrons (MLP) neural network architecture, but the method can also be adapted to other types of architectures.

Two objective functions are used in BFS. The first objective, denoted as *f*_1_, is the cross-entropy:

$$\begin{array}{c} f_{1}\left(\theta \right)=-\frac{1}{N}\sum_{i=1}^{N}\sum_{k=1}^{K}Y_{ik}\;\text{log}\left({\tilde{Y}}_{ik}\left(X_{i},\theta \right)\right). \end{array}$$ (1)

where *Y*_*ik*_ and ${\tilde{Y}}_{ik}$ the *k*-th component of the class vector *Y*_*i*_ and predicted vector $\tilde{Y_{i}}$ respectively. The second objective is the sparse group lasso regularization term [19] that allows the removal of individual connections and neurons from the network.

The idea is to consider sparsity at the level of groups of connections to force all outgoing connections from a single neuron to be simultaneously set to zero. More precisely, the aim is to consider two non-overlapping groups of connections that correspond to the sparsity groups:

1. *Groups representing the input layer G*_*input*_
  An element *g* ∈ *G*_*input*_ is a vector that represents the weights of all outgoing connections from a neuron in the input layer: $G_{input}=\left\{g\in R^{H_{1}}/g=\left(w_{i1}^{1},\ldots ,w_{iH_{1}}^{1}\right),i=1,\ldots ,H_{0}\right\}$;
2. *Groups representing the hidden layers G*_*hidden*_
  An element *g* ∈ *G*_*hidden*_ is a vector that corresponds to the weights of all outgoing connections from a neuron in the hidden layers: $G_{hidden}=\left\{g\in R^{H_{l+1}}/g=\left(w_{i1}^{l+1},\ldots ,w_{iH_{l+1}}^{l+1}\right),l=1,\ldots ,L-1,i=1,\ldots ,H_{l}\right\}$.

Let *G* = *G*_*input*_ ∪ *G*_*hidden*_. The second objective, denoted as *f*_2_, is the sparse group lasso regularization term:

$$\begin{array}{c} f_{2}\left(\theta \right)=\sum_{g\in G}\sqrt{\#g}{\;||g||}_{2}+\sum_{l=1}^{L}\sum_{i=1}^{H_{l}}\left|\right|\theta_{i}^{l}{\left|\right|}_{1}, \end{array}$$ (2)

where #*g* represents the dimension of the vector *g* and ||*g*||_2_ the Euclidean norm of $\theta_{i}^{l}$ and $\left|\right|\theta_{i}^{l}\left|\right|$ its L1 norm. Note that this objective encourages sparsity at both the individual and group levels. In the sequel of this section, the optimization approach used in this method is presented and then, the initialization and training steps are explained.

### Optimization approach

This work proposes a new approach for feature selection based on architecture optimization, using a constrained multiobjective gradient descent by Lin et al. [9] with two major modifications to overcome two limitations of their proposed work. Two kinds of normalization are performed during the search process in order to take into account the large difference in scales between the two objective functions. Moreover, the regions are defined differently, using only 2 constraints instead of K. This leads to fewer constraints in the optimization problem, making the problem faster to solve, especially for large values of K.

**Fig 1:**
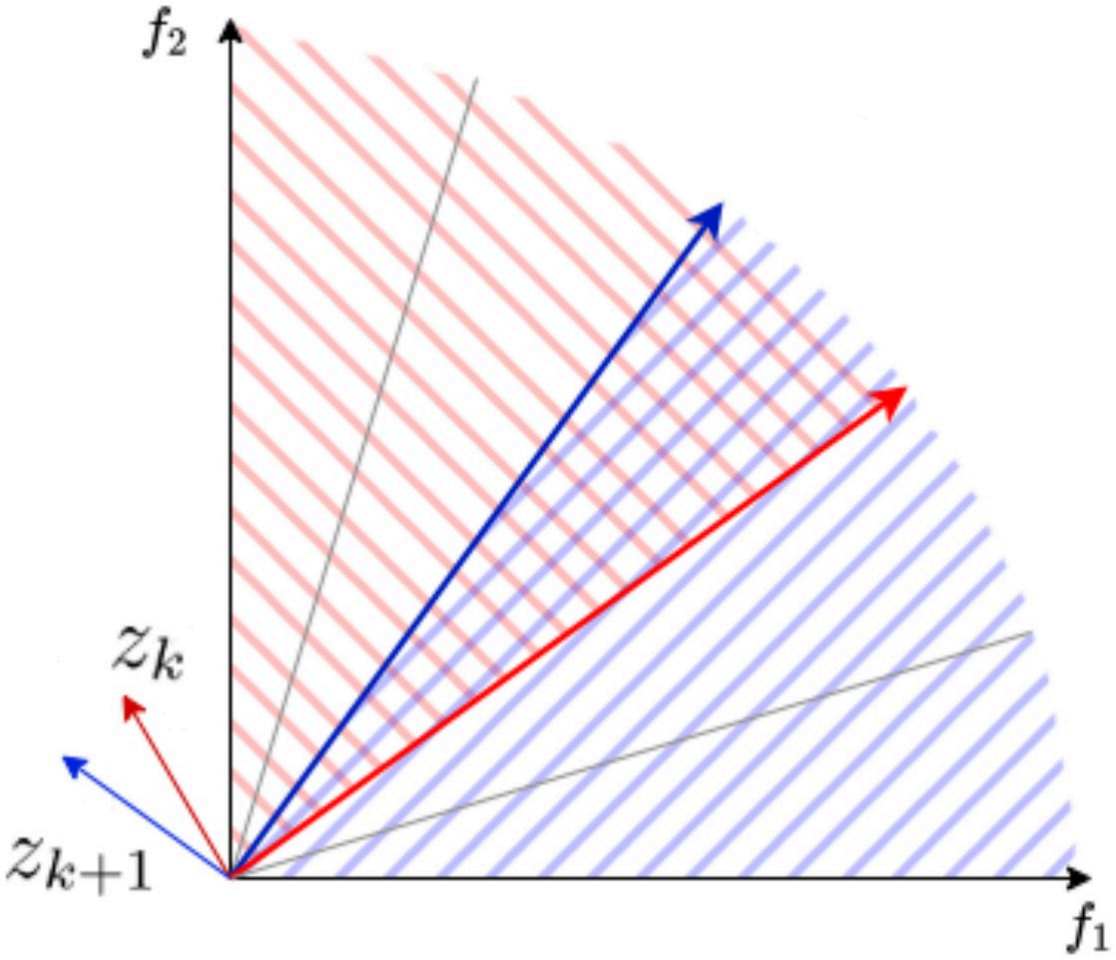
The region $\Psi_{k}=\left\{v\in R_{+}^{2}|v.z_{k}\geq 0\;\text{and}\;v.z_{k+1}\leq 0\right\}$.

Before running the algorithm, the number of regions K must be chosen and the objective space is divided accordingly into K different regions, denoted by Ψ_*k*_ (1≤k≤K) such that $\Psi_{k}=\left\{v\in R_{+}^{2}\right|v.z_{k}\geq 0$ and *v*.*z*_*k*+1_ ≤ 0} where *z*_*k*_ and *z*_*k*+1_ are the normal vector starting from the origin with respect to the vectors delimiting the region as shown in Fig 1. The biobjective problem with respect to the *k*-th region can now be written as follows:

$$\begin{array}{c} \text{min}\;F(\theta )\\ s.t\;G_{1}^{k}(\theta )=-F(\theta ).z_{k}\leq 0\\ \text{and}\;G_{2}^{k}(\theta )=F(\theta ).z_{k+1}\leq 0 \end{array}$$ (3)

With *F*(*θ*) = (*f*_1_(*θ*), *f*_2_(*θ*)) where *f*_1_ and *f*_2_ are the two objective functions defined above.

In practice, as the two objective functions are of very different scales, normalization is required.

**Fig 2:**
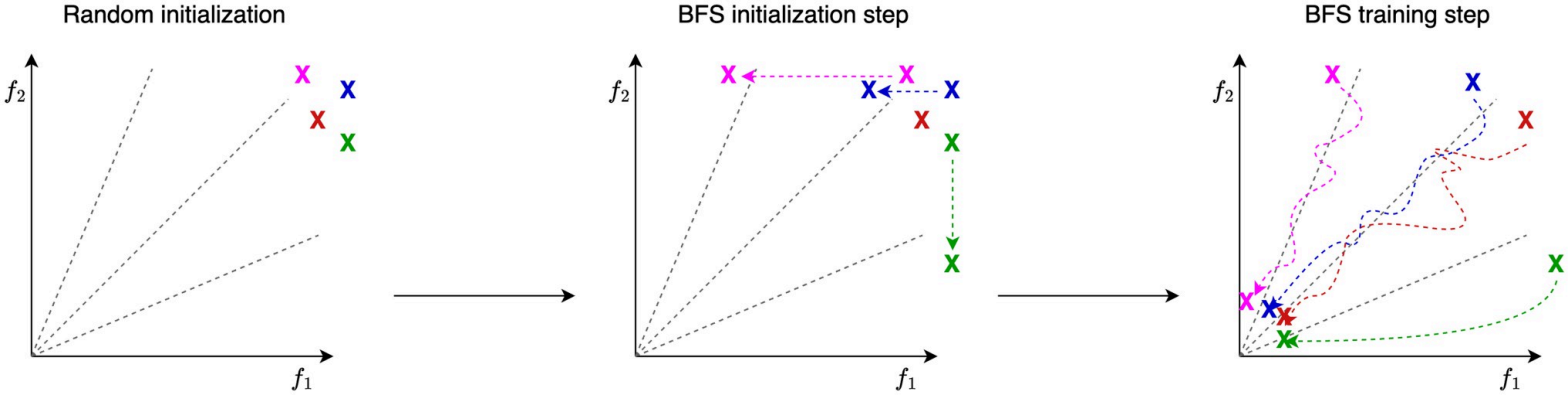
Figure showing the different steps of the BFS method: Initial solutions (left), initialization step (middle) and training step (right).

The proposed method called BFS for Biobjective Feature Selection consists of two steps: an initialization step that leads to dispersed initial solutions and a training step that provides a Pareto front. Fig 2 illustrates the two steps.

#### Initialization step

The first step is to initialize the networks. This step is very important as it allows initial solutions to be dispersed in the objective space. This leads to well-representative solutions. For this, the algorithm starts from a random solution *θ*, and for each 1≤k≤K, gradient descent is performed to get a solution inside the region Ψ_*k*_ or at least as close to it as possible. Let $I_{∊}\left(\theta \right)=\left\{j|G_{j}^{k}\left(\theta \right)\geq -\varepsilon ,j=1,2\right\}$ be the set of active constraints, where *ε* is a threshold added to avoid solutions to remain on the region boundaries. The optimal descent direction *d*_0_ and the temporary variable *γ*_0_ are computed by solving the following problem:

$$\begin{array}{c} (d_{0},\gamma_{0})=\text{arg}\;\underset{d\in {\mathbb{R}}^{n},\gamma \in \mathbb{R}}{\text{min}}\;\gamma +\frac{1}{2}||d|{|}^{2}\text{s.t}\\ s.t\;\nabla G_{j}^{k}(\theta ).d\leq \gamma ,j\in I_{\epsilon }(\theta ) \end{array}$$ (4)

**Fig 3:**
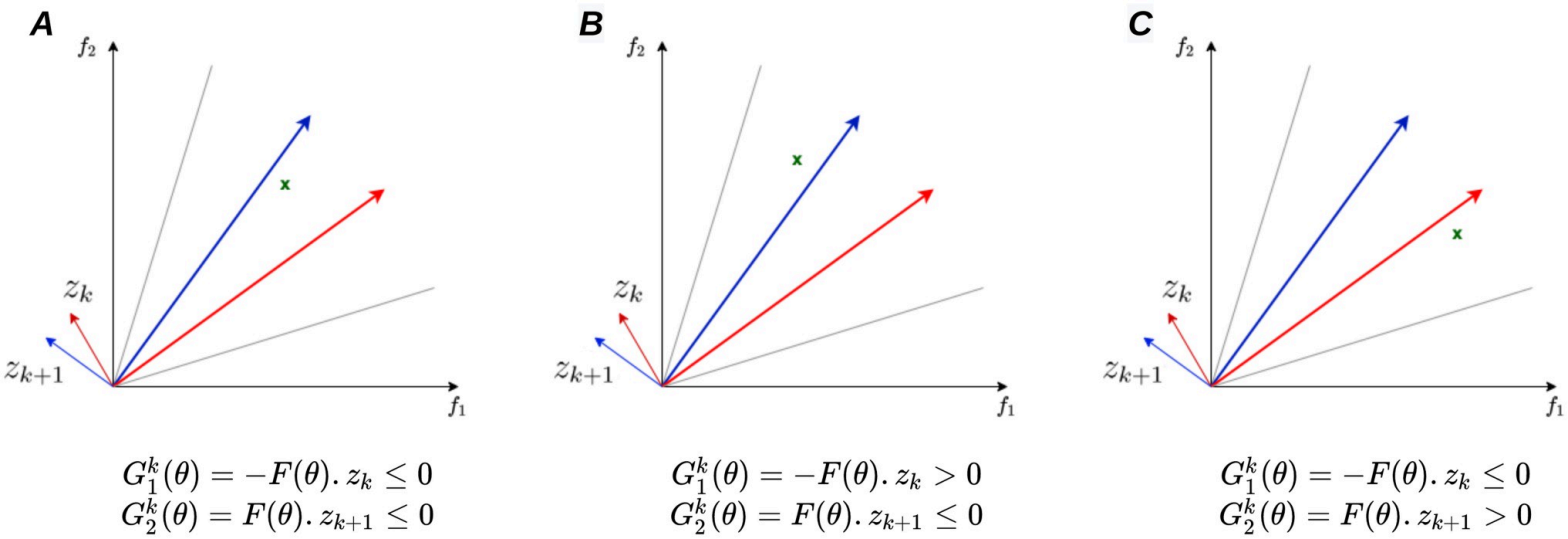
The different possible scenarios for a solution at each iteration. (A) the solution is in its region, (B) the solution is outside the region from the left and (C) the solution is outside its region from the right.

As can be seen in Fig 3, there can be at most one active constraint, i.e. #*I*_*ϵ*_(*θ*) ≤ 1:

1. If #*I*_*ϵ*_(*θ*) = 0, the solution is in its region, and there is no need to pursue the gradient descent (Fig 3(A)).
2. If #*I*_*ϵ*_(*θ*) = 1 the solution is not in its region, gradient descent is performed in order to minimize the degree of violation of the active constraint (Fig 3(B) and 3(C)).

**Fig 4:**
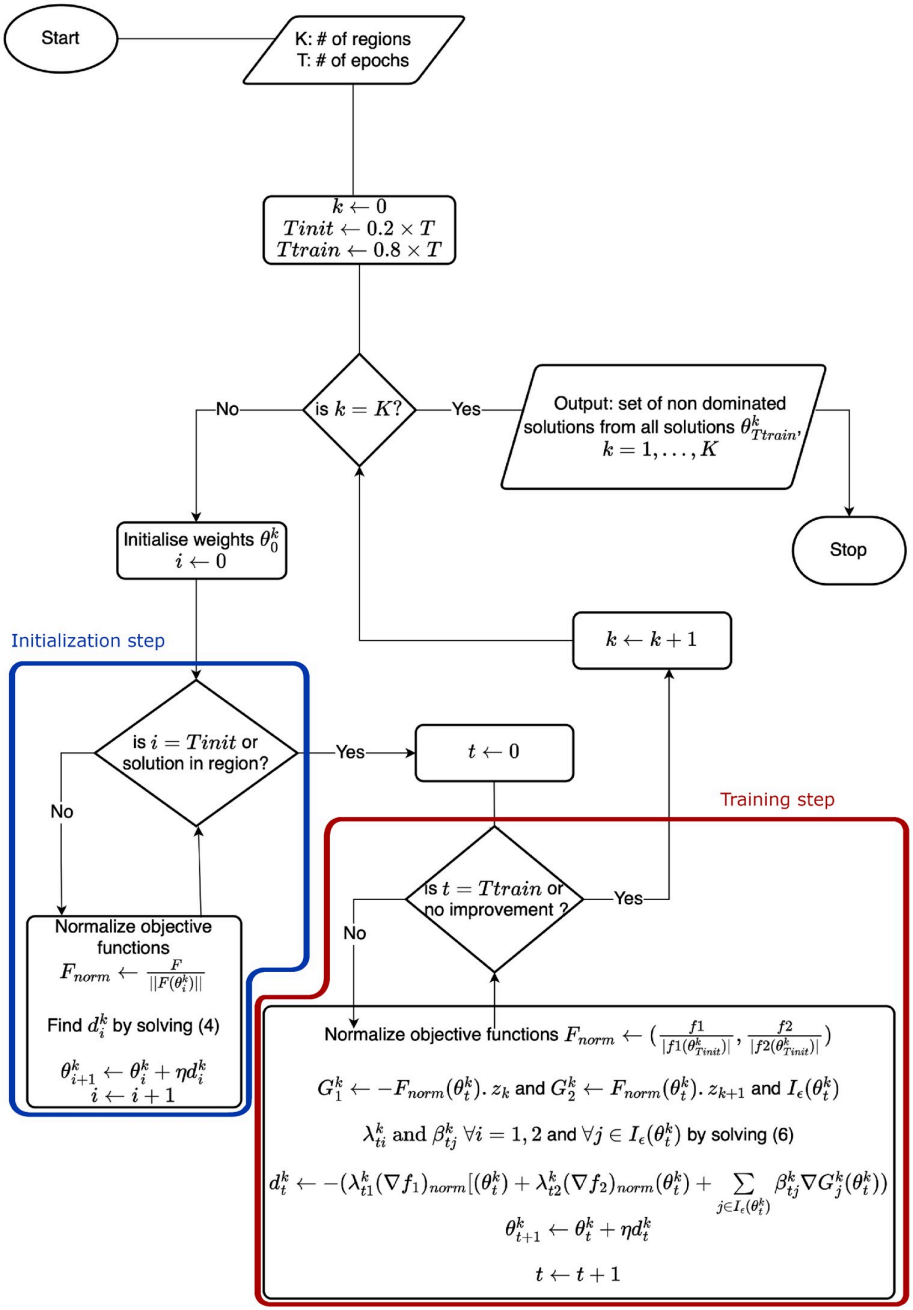
Detailed workflow of the BFS algorithm. In the blue frame are shown the steps used during the initialization steps. Once the solution is in its region or the maximum number of iterations is reached, it will move to the red frame steps. The red frame corresponds to the training steps, which will keep running until the maximum epochs is reached or no more improvements are detected for 10 consecutive iterations.

In this step, as shown in the diagram in Fig 4, normalization consists of dividing each objective function by the norm-2 of the vector composed of the objective functions. The termination criteria for this step are either the region is reached or 20% of the total number of iterations have passed.

#### Training step

After the initial solution is reached, each network is trained using a constrained multiple-gradient descent algorithm. The optimal descent direction d* can be obtained by solving the following problem:

$$\begin{array}{c} \left(d^{*},\gamma^{*}\right)=\text{arg}\underset{d\in R^{n},\gamma \in R}{\text{min}}\gamma +\frac{1}{2}{\left||d|\right|}^{2}\\ \begin{array}{cc} s.t\;\nabla f_{i}\left(\theta \right).d & \leq \gamma ,\;i=1,2.\\ \nabla G_{j}^{k}\left(\theta \right).d & \leq \gamma ,\;j\in I_{\epsilon }\left(\theta \right), \end{array} \end{array}$$ (5)

where γ* is a temporary variable, ∇*f*_*i*_(*θ*) the gradient of the objective function *i* and $\nabla G_{j}^{k}\left(\theta \right)$ the gradient of the constraint *j*. However, because of the high number of parameters found in neural networks, it is more efficient to solve the following related dual problem (6). For more details on how to obtain the dual, please refer to S1 Appendix (Remark 3).

$$\begin{array}{c} \underset{\lambda_{i},\beta_{j}}{\text{max}}\;-\frac{1}{2}||\sum_{i=1}^{2}\lambda_{i}\nabla f_{i}(\theta )+\sum_{j\in I_{\epsilon }(\theta )}\beta_{j}\nabla G_{j}^{k}(\theta )|{|}^{2}\\ s.t.\;\sum_{i=1}^{2}\lambda_{i}+\sum_{j\in I_{\epsilon }(\theta )}\beta_{j}=1,\\ \lambda_{i}\geq 0,\;\beta_{j}\geq 0,\forall i=1,2,\;\forall j\in I_{\epsilon }(\theta ), \end{array}$$ (6)

where λ_*i*_ and *β*_*j*_ are the Lagrange multipliers. The descent direction d* is then calculated as

$$\begin{array}{c} d^{*}=-(\lambda_{1}\nabla f_{1}\left(\theta \right)+\lambda_{2}\nabla f_{2}\left(\theta \right)+\sum_{j\in I_{\epsilon }\left(\theta \right)}\beta_{j}\nabla G_{j}^{k}\left(\theta \right)). \end{array}$$ (7)

In this step, as shown in the diagram in Fig 4, the normalization consists of dividing each objective function by the absolute value of the objective function obtained at the end of the initialization for the network being trained. The algorithm stops when a number of epochs is reached or when there is no more improvement for 10 successive epochs.

The BFS method is summarized in Fig 4.

## Experiments

BFS is compared to four different algorithms: L1-NN, SGL-NN, MOO-MTL, and BFS init only. L1-NN represents an MLP with a scalarized objective function that combines the cross-entropy and the L1 regularizer. SGL-NN is also a mono-objective MLP with a scalarized objective function combining the cross-entropy and the Sparse Group Lasso (SGL) regularizer as in [5]. MOO-MTL is an MLP based on an unconstrained multiobjective optimization as in [8]. BFS init only combines BFS and MOO-MTL: the networks are initialized using the BFS initialization step and then the unconstrained multiobjective optimization from MOO-MTL is used for the training step. ParetoMTL comparison was omitted as it was impossible to generate results because of a high running time. See Section 1.3 in S2 Appendix for more details.

For each method, a combination of random search and cross-validation are used to identify the optimal set of hyperparameters. In order to get a large number of solutions, the objective space for BFS is split into 100 regions. For a fair comparison, each of the methods mentioned above was run 100 times using the best hyperparameters found. Details on the hyperparameter choices and their search range can be found in Section 1.2 in S2 Appendix.

To assess the quality of the obtained solutions in terms of classification, feature selection, and sparsity, the following metrics were used:

- **Classification metrics:** used to evaluate the performance of a model. Four metrics will be used in this work. First, the classification **accuracy**, which is an indicator of how often the classifier is correct overall. Second, **recall**, which shows whether a model can find all objects of the target class. Then, **precision**, which indicates how often the model is correct when predicting the target class. Finally, the **F1-score**, which is the harmonic mean of the recall and precision scores, and serves to assess the overall performance of a model.
- **FS score:** to evaluate the feature selection capacity of a method. Let *G*_*imp*_ be the set of outgoing connection weight vectors of the input neurons corresponding to the relevant features and *G*_*input*_ the set of outgoing connection weight vectors of all input neurons. The *FS* score is defined similarly to the Informative Features Weight Ratio (IFWR) score, introduced in [14], as follows:
  $$\begin{array}{c} FS=\frac{\sum_{g\in G_{imp}}{\left||g|\right|}_{1}}{\sum_{g\in G_{input}}{\left||g|\right|}_{1}}. \end{array}$$
  The higher the *FS* score, the better the feature selection capacity of the method.
  In addition, to get the importance and rank each feature, the norm-1 value of the outgoing weights for each input neuron is computed. In other words, for each feature *g*, where *g* ∈ *G*_*input*_, the following score is calculated:
  $$\begin{array}{c} score\left(g\right)={\left||g|\right|}_{1}. \end{array}$$
  The higher *score*(*g*) is, the more important the feature *g* is.
- **Sparsity score:** All weights with an absolute value smaller than 10^−5^ are considered to be equal to 0. Let *θ*_*null*_ be the set of weights equal to 0 and *θ* the set of all weights in a network. The sparsity score *S* is defined as follows:
  $$\begin{array}{c} S=\frac{|\theta_{null}|}{\left|\theta \right|}. \end{array}$$
  The higher the score, the more sparse the network.

Using the above described metrics, BFS is compared to the previously described state-of-the-art methods on artificial and real HDLSS datasets.

### Artificial datasets

AD1 and AD2, two artificial datasets of different difficulty levels, were considered. They were generated using the scikit-learn [40] package. Details about each dataset are provided in Table 1. Section 1.1 S2 Appendix, contains more information for reproducing the datasets. The AD1 dataset is a binary classification problem and is relatively easy since it contains only 2 classes. The AD2 dataset is harder as it is a multiclass classification problem with 3 classes, and the ratio of informative features to noise data is lower than in the AD1 dataset. In both datasets, the informative features follow a standard normal distribution, the rest of the features are just noise data following a uniform distribution. In order to simulate HDLSS data, the number of train instances was set to be lower than the total number of features in each dataset.

**Table 1:** Table summarizing the characteristics of the artificial datasets used.

Dataset	Train instances	Test instances	Important features	Total features	Classes
AD1	900	2100	50	2000	2
AD2	900	2100	100	3000	3

**Fig 5:**
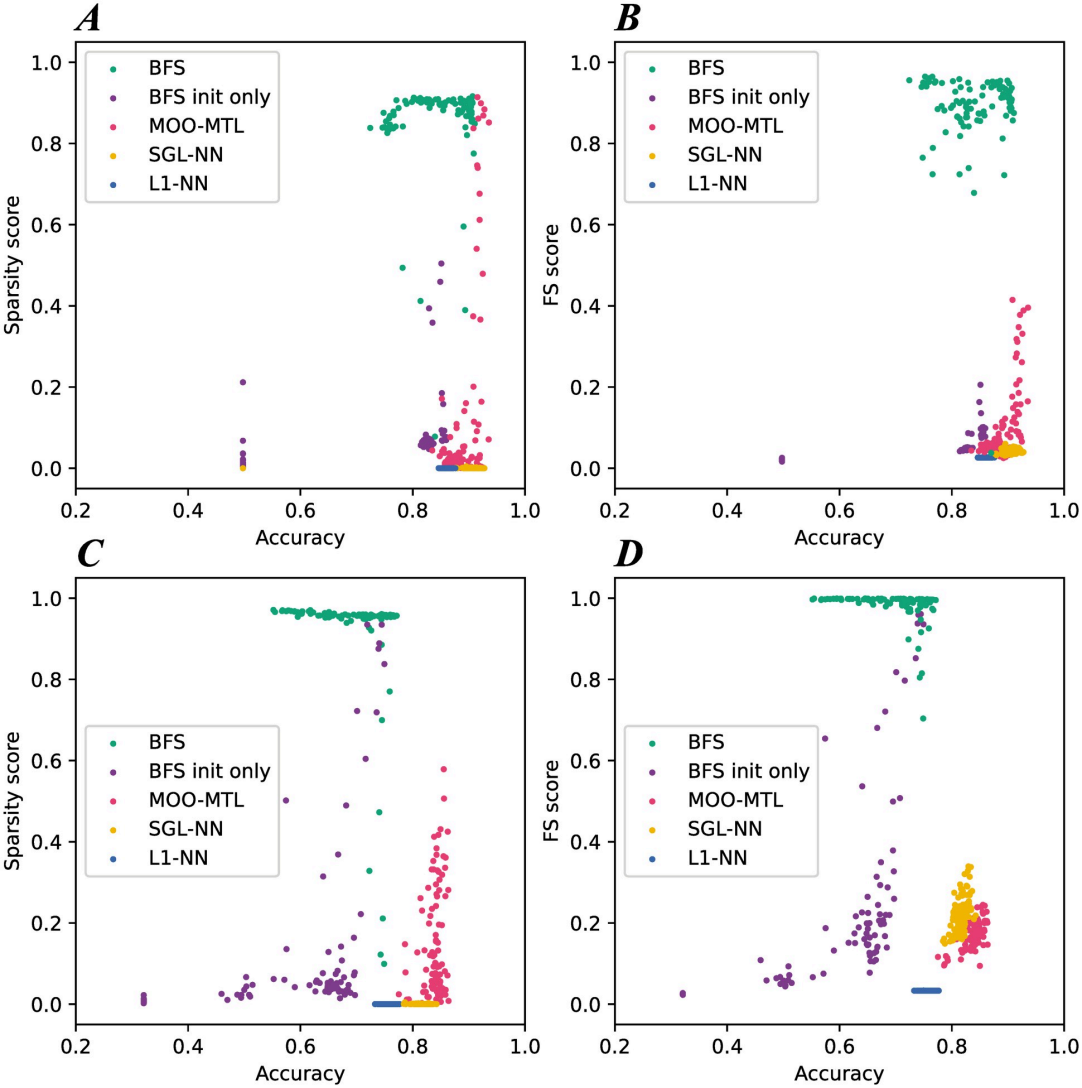
Summary of the results obtained on artificial datasets AD1 (subfigures A and B) and AD2 (subfigures C and D).

Each algorithm was run 10 times. In each run, 100 neural networks were constructed. Table 2 summarizes the results obtained on the artificial datasets AD1 and AD2. The 5 methods are first compared by evaluating 6 metrics: the accuracy, precision, recall and F1-score as the classification metrics, the *FS* score as the feature selection metric and the *S* score as the sparsity score. The values reported in Table 2 correspond to the mean of the highest values obtained in each run. For the classification metrics, the network with the highest accuracy among the 100 network in each run, is used to calculate the mean of each metric. For the *FS* and *S* scores, the network with the highest *FS* score and highest *S* score respectively are used. In the case of AD1, BFS, MOO-MTL and SGL-NN all have similar high accuracy, precision, recall and F1 score, around 0.900. L1-NN and BFS init only have a lower accuracies around 0.820. The other classification metrics all have the same score as the accuracy. BFS has the highest *FS* and *S* sores with 0.970 and 0.916 respectively followed closely by BFS init only with a score of 0.907 for FS and 0.871 for the sparsity. MOO-MTL comes in third with low FS score and sparsity, 0.202 and 0.344 respectively. Finally, with both L1-NN and SGL-NN, neither feature selection or network sparsification occurs as for both methods the scores are low. As for AD2, BFS and BFS init only have the highest *FS* score, 0.998 and a very similar *S* score, 0.96 and 0.95 respectively. For BFS, all classification metrics are around 0.770. For BFS init only, all classification metrics are around 0.630, the lowest among all methods. BFS’ accuracy is slightly lower than the highest accuracy obtained on this dataset which is 0.807. This accuracy is obtained by MOO-MTL. However, MOO-MTL performs poorly in terms of feature selection and sparsification, with a *FS* score of 0.229 and a *S* score of 0.471 meaning prediction was based on the wrong features. SGL-NN has the same accuracy as BFS, 0.763 but also performs poorly on feature selection and sparsity with 0.329 and 0.009 on the *FS* and *S* scores respectively. As for L1-NN, the models have a low accuracy, 0.687 and no feature selection (0.034) and no sparsity (0.000) at all. In addition to the different metrics, Fig 5, shows the Pareto front and solution dispersion for one run for each algorithm (100 networks). Fig 5(A) and 5(B) shows the sparsity score and the FS score respectively, along with the test accuracy, on AD1. BFS has the most complete front with the solutions being dispersed in the objective space. BFS init only manages to have very few solutions that are on the Pareto front with the rest all having low sparsity and few relevant features selected only. With MOO-MTL, the same problem occurs except that no solution reaches a high feature selection score which is in concordance with the results obtained on the 10 runs. As for SGL-NN and L1-NN, there is no Pareto front that can be seen as all solutions for both methods are gathered in one place, and no diversity in the solutions can be seen. Fig 5(C) and 5(D) show the sparsity score and the FS score respectively, along with the test accuracy, on AD2 for all solutions returned by the 5 methods. Similarly to AD1, BFS is the method with the most complete Pareto front. BFS init only has a few solutions with high values in both feature selection and sparsification while MOO-MTL solutions are gathered, and the method obtains low *S* and *FS* scores. SGL-NN and L1-NN have no diversity in the solutions like for AD1. In summary, replacing L1 with SGL in a mono objective network is not enough to enhance the ability of neural networks to select features. This study highlights the significance of multiobjective optimization in creating networks that are both accurate and sparse while also relying on the correct features.

**Table 2:** Table summarizing the obtained metrics on the artificial datasets. Metrics shown are the averages computed from 10 runs.

Method	BFS	BFS init only	MOO-MTL	L1-NN	SGL-NN
**AD1**					
Accuracy	0.912 ± 0.0023	0.823 ± 0.110	0.904 ± 0.011	0.825 ± 0.012	0.885 ± 0.006
Precision	0.912 ± 0.003	0.801 ± 0.185	0.905 ± 0.011	0.827 ± 0.012	0.885 ± 0.006
Recall	0.912 ± 0.003	0.823 ± 0.110	0.904 ± 0.011	0.825 ± 0.012	0.885 ± 0.006
F1 score	0.912 ± 0.003	0.806 ± 0.159	0.904 ± 0.011	0.825 ± 0.012	0.885 ± 0.006
*FS* score	0.970 ± 0.006	0.907 ± 0.234	0.202 ± 0.092	0.027 ± 0.000	0.050 ± 0.005
*S* score	0.916 ± 0.004	0.871 ± 012	0.344 ± 0.344	0.000 ± 0.000	0.003 ± 0.001
**AD2**					
Accuracy	0.774 ± 0.008	0.627 ± 0.085	0.807 ± 0.006	0.687 ± 0.024	0.763 ± 0.022
Precision	0.777 ± 0.008	0.634 ± 0.086	0.808 ± 0.006	0.689 ± 0.024	0.765 ± 0.022
Recall	0.775 ± 0.008	0.630 ± 0.082	0.808 ± 0.006	0.688 ± 0.024	0.764 ± 0.022
F1 score	0.774 ± 0.0083	0.611 ± 0.107	0.807 ± 0.006	0.689 ± 0.024	0.763 ± 0.022
*FS* score	0.998 ± 0.000	0.998 ± 0.001	0.229 ± 0.043	0.034 ± 0.000	0.329 ± 0.038
*S* score	0.964 ± 0.003	0.946 ± 0.004	0.471 ± 0.210	0.000 ± 0.000	0.009 ± 0.003

#### Real datasets

For these experiments, two real text datasets were used: BASEHOCK and PCMAC, introduced in [41]. The features in these datasets represent real and noise variables. In order to mimic HDLSS data, only 20% of the dataset was used for training, and the rest was used as a test dataset. Both datasets are used for feature selection purposes. BASEHOCK contains 2 classes, 4862 features and 1993 samples. The features are all sparse discrete variables with a majority of values being equal to 0. 994 samples belong to class 0 and 999 to class 1. As for PCMAC, the dataset is also composed of 2 classes with 1943 samples and 3289 sparse discrete features. The dataset is also balanced with 982 samples of class 0 and 961 of class 1. The relevant characteristics of the datasets used are summarized in Table 3. Similarly to artificial datasets, in addition to the classification metrics, the sparsity and feature selection capacity of each method are evaluated.

**Table 3:** Table summarizing the characteristics of the real datasets.

Dataset	Train size	Test size	Features	Classes
Basehock	597	1396	4862	2
PCMAC	582	1361	3289	2

Each method was also run 10 times, and the reported metrics in Table 4 represent the mean for the best network per iteration of each metric for each method, similarly as for artificial datasets. In the case of real datasets, only the classification metrics and the *S* score are calculated as the relevant features are not known and the feature selection ability will be evaluated later. For BASEHOCK, all methods have a similar accuracy ranging from 0.898 for L1-NN and SGL-NN to 0.893 for M00-MTL and finally to 0.874 for BFS except for BFS init only who has an accuracy of 0.735. The other classification metrics are all the same as the accuracy for each method. However, the main difference between the methods lies in the *S* score. With a score of 0.97 and 0.98, respectively, BFS and BFS init only give the most sparse networks. MOO-MTL comes after with 0.848 sparse network. Finally, as in the case of the previous datasets, L1-NN and SGL-NN can not achieve any sparsification, which can be seen with the null score obtained. As for PCMAC, the results follow the same tendency, with a close accuracy between the different methods ranging from 0.791 for BFS to 0.815 for MOO-MTL and higher *S* score for BFS and BFS init only than the other methods. The lowest accuracy was once again for BFS init only The other classification metrics take the same values as the accuracy for each method.

**Table 4:** Table summarizing the obtained metrics on the real datasets. Metrics shown are the averages computed from 10 runs.

Method	BFS	BFS init only	MOO-MTL	L1-NN	SGL-NN
**BASEHOCK**					
Accuracy	0.874 ± 0.008	0.735 ± 0.0065	0.893 ± 0.002	0.898 ± 0.009	0.898 ± 0.009
Precision	0.877 ± 0.008	0.755 ± 0.092	0.896 ± 0.003	0.898 ± 0.009	0.900 ± 0.009
Recall	0.874 ± 0.008	0.735 ± 0.085	0.894 ± 0.002	0.898 ± 0.009	0.897 ± 0.009
F1 score	0.874 ± 0.008	0.731 ± 0.084	0.894 ± 0.002	0.898 ± 0.009	0.897 ± 0.009
*S* score	0.970 ± 0.002	0.982 ± 0.003	0.848 ± 0.285	0.000 ± 000	0.000 ± 0.000
**PCMAC**					
Accuracy	0.791 ± 0.005	0.694 ± 0.036	0.816 ± 0.015	0.808 ± 0.015	0.815 ± 0.016
Precision	0.792 ± 0.005	0.699 ± 0.035	0.816 ± 0.016	0.808 ± 0.016	0.816 ± 0.016
Recall	0.792 ± 0.005	0.699 ± 0.036	0.816 ± 0.015	0.808 ± 0.016	0.815 ± 0.016
F1 score	0.791 ± 0.005	0.691 ± 0.037	0.816 ± 0.015	0.808 ± 0.016	0.815 ± 0.016
*S* score	0.979 ± 0.003	0.949 ± 0.007	0.866 ± 0.237	0.000 ± 0.000	0.000 ± 0.000

**Fig 6:**
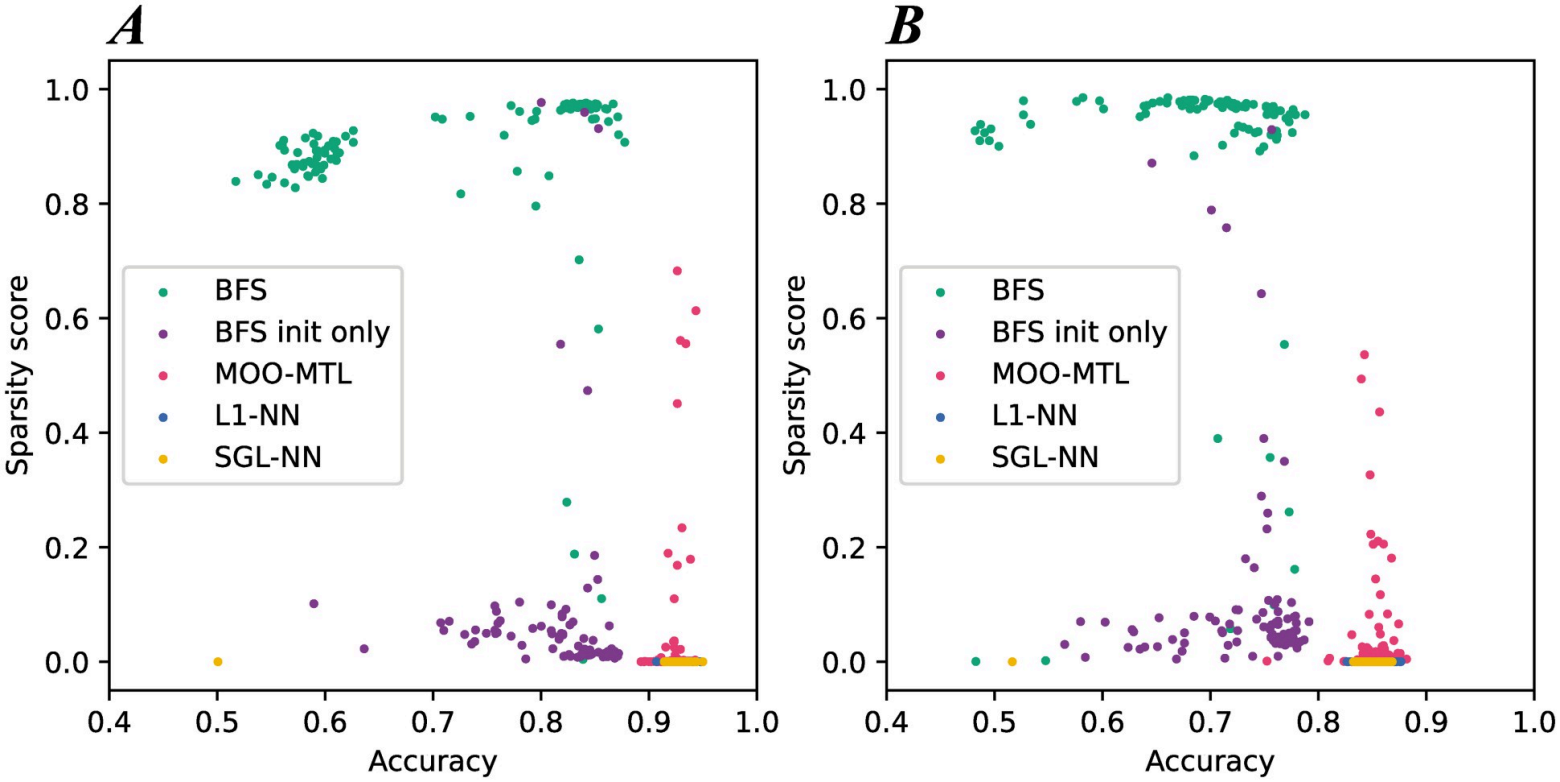
Summary of the sparsity evaluation results on the real datasets. (A) shows the sparsity and test accuracy of the solutions obtained with the different methods on the BASEHOCK dataset. (B) shows the sparsity and test accuracy of the solutions obtained with the different methods on the PCMAC dataset.

The Pareto front and diversity of the obtained solutions on 1 run are shown in Fig 6. Fig 6(A) shows the obtained solutions on BASEHOCK, whereas Fig 6(B) shows the results obtained on PCMAC. For BASEHOCK, BFS is the only method with a complete Pareto front and diverse solutions. BFS init only has only 3 out of the 100 solutions with the rest of the solutions towards the bottom of the space as can be seen in Fig 6. Similarly, for MOO-MTL, few solutions only can be differentiated from the rest of the solutions with a very low sparsity. SGL-NN and L1-NN have no diversity in the solutions as they are all stacked at the same position. PCMAC results follow the same pattern as BASEHOCK results which is also in concordance with the different calculated metrics. BFS has a high diversity between the solution in BFS. For BFS init only and MOO-MTL, only few solutions have a high sparsity with the others all gathered in the lower part of the objetcive space. As for SGL-NN and L1-NN, they have the same problem as in the other datasets: no sparsity occurs in networks trained with these methods.

**Fig 7:**
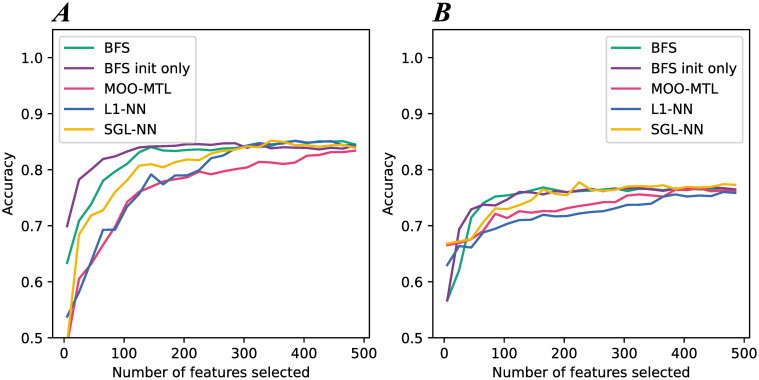
Summary of the feature selection evaluation results on the real datasets. (A) shows the mean test accuracy obtained when training the classification algorithms based on the number of features selected by the different methods to compare on the BASEHOCK dataset. (B) shows the mean test accuracy obtained when training the classification algorithms based on the number of features selected by the different methods to compare on the PCMAC dataset.

When dealing with real datasets, relevant features are usually not known beforehand, unlike in artificial datasets. Therefore, the ability of a method to perform feature selection using the *FS* score introduced earlier is not possible. Hence, to assess feature selection capacity, each algorithm will be considered as a filter method to select a subset of features. Then, the selected features will be used as input to train a classification algorithm. If the selected features are relevant, the classification algorithm’s accuracy will increase rapidly before stabilizing. In this protocol, the solution with the highest accuracy for each method and each dataset is first selected. The features are then ranked using the score described earlier using the norm-1 and the *N* most highly ranked features are selected. Afterwards, the selected features are used as input to three traditional classification algorithms: Random Forest (RF), Extreme Gradient Boosting (XGB), and Support Vector Classification (SVC). The mean test accuracy obtained with all three methods is then reported. This procedure is repeated by incrementing the number of chosen features *N*, retraining the three algorithms, and calculating the mean test accuracy again. The procedure is stopped when there is no longer any significant improvement in the test accuracy. The results are displayed in Fig 7.

Fig 7(A) shows the accuracy obtained when the number of selected features is varied on the BASEHOCK dataset. Fig 7(B) shows the accuracy obtained when the number of selected features is varied on the PCMAC dataset. Recall that the results shown are for one iteration only. The focus in both datasets was on the first 500 features only since no significant accuracy improvement was observed beyond that with any method. For BASEHOCK, with 5 features used to train the algorithms, BFS init only starts with the highest accuracy, 0.7, followed by BFS, 0.65, and then L1-NN, MOO-MTL, and SGL-NN with a starting accuracy of 0.55 and below. All methods will converge to an accuracy around 0.85 after adding 500 features. However, when adding only the first 130 ranked features, BFS and BFS init are the only methods that reach it. 300 additional selected features by L1-NN and SGL-NN need to be added to reach this same accuracy, and the first 500 selected features from MOO-MTL are needed to obtain this same accuracy. As for PCMAC, after adding 5 features, SGL-NN and MOO-MTL start with an accuracy of 0.65, L1-NN with an accuracy of 0.63, and BFS and BFS init only with an accuracy of 0.57. However, it then requires training with less than 100 features selected with BFS to reach an accuracy of 0.75. With BFS init only, around 120 features are needed to reach this same accuracy, and 170 features are needed with SGL-NN. MOO-MTL and L1-NN select the least relevant features as the first 370 selected features are needed with MOO-MTL and all 500 features selected with L1-NN to reach the same accuracy as with the previous methods.

While the results show that BFS manages to get higher results in terms of feature selection and sparsity, a statistical analysis was conducted to check if there is a significant improvement and confirm the obtained findings. For each dataset, 10 values, the best from each iteration, was used. The tests were performed three times: once one the accuracy metric, once on the sparsity metric and once on the feature selection metric. In addition, in order to decide the test to use, the safe use of parametric tests conditions, that is independence, normality and homoscedasticity are tested. The independence condition is verified as for each method and dataset, the 10 run are independent. For the normality condition, the Shapiro-Wilk test was run and the obtained p-values are reported in Table 5 for all three metrics. The p-values are all smaller that *α* = 0.05, except for the *FS* score in the case of MOO-MTL, meaning that the normality condition is not satisfied therefore, the use of non parametric tests is more appropriate.

**Table 5:** Table showing the Shapiro-Wilk p-values for the normality condition verification.

	BFS	BFS init only	MOO-MTL	L1-NN	SGL-NN
Accuracy	3.10 × 10^−5^	5.90 × 10^−4^	2.39 × 10^−6^	7.97 × 10^−6^	1.18 × 10^−4^
*S* score	1.20 × 10^−6^	3.47 × 10^−10^	1.87 × 10^−5^	7.70 × 10^−9^	5.82 × 10^−6^
*FS* score	2.70 × 10^−5^	8.54 × 10^−5^	1.52 × 10^−1^	9.37 × 10^−5^	2.12 × 10^−5^

As there are only 5 methods and 10 runs for each methods, separate Wilcoxon tests were performed between the different methods for each dataset, with BFS as the control as suggested in [42]. The obtained p-values for all metrics are presented in Tables 6–8 for the accuracy, *S* score and *FS* score respectively. Two methods are considered different on a metric and for a dataset if the corresponding p-value is less than *α* = 0.05. For the accuracy, SGL-NN and BFS as well as L1-NN have equal accuracies as the p-value is greater than 0.05 for all datasets. For BFS init only and MOO-MTL, the accuracies are different when compared to BFS as the p-value is less than 0.05 for all datasets. As for the sparsity and feature selection, the difference between the values obtained with BFS and MOO-MTL, SGL-NN and L1-NN is significant as for all datasets, the p-value is less than 0.05 for both metrics. However, when compared to BFS init only, the hypothesis that the two methods are equal in terms of sparsity can not be rejected for the BASEHOCK dataset as the p-value is 0.084 which is greater than 0.05. Similarly, the hypothesis that BFS and BFS init only are equal in terms of feature selection can not be rejected as the p-values are 0.08 and 0.23 on AD1 and AD2 respectively. These tests show that BFS improves the sparsity and feature selection abilities of a network. As for BFS init only, while both sparsity and feature selection abilities are the same as in BFS, the accuracy is not and there is no real diversity in the obtained solutions.

**Table 6:** Table with the p-values obtained with the different Wilcoxon tests performed on the accuracy metric.

	AD1	AD2	BASEHOCK	PCMAC
BFS vs BFS init only	0.001	0.002	0.003	0.002
BFS vs MOO-MTL	0.001	0.049	0.001	0.037
BFS vs SGL-NN	0.280	0.130	0.770	0.490
BFS vs L1-NN	0.920	0.084	0.560	0.084

**Table 7:** Table with the p-values obtained with the different Wilcoxon tests performed on the sparsity metric.

	AD1	AD2	BASEHOCK	PCMAC
BFS vs BFS init only	0.049	0.001	0.084	0.001
BFS vs MOO-MTL	0.001	0.001	0.002	0.006
BFS vs SGL-NN	0.002	0.002	0.002	0.002
BFS vs L1-NN	0.002	0.000	0.002	0.002

**Table 8:** Table with the p-values obtained with the different Wilcoxon tests performed on the feature selection metric.

	AD1	AD2
BFS vs BFS init only	0.080	0.230
BFS vs MOO-MTL	0.002	0.002
BFS vs SGL-NN	0.002	0.002
BFS vs L1-NN	0.002	0.002

## Discussion

In this work, BFS, a novel constrained biobjective optimization method to perform feature selection and network sparsification has been presented. This approach proposes a new formulation for the constrained multiobjective optimization that is faster and easier to use. In fact, by limiting the number of constraints needed at every iteration of the gradient descent step, the running time of the algorithm is decreased and it is possible to split the objective space into many regions to diversify the solutions. The latter is a downside of the ParetoMTL method as it is impossible to run it on 100 regions (refer to Section 1.3 S2 Appendix for details). Using the dual method and the cvxopt solver, which takes less than a fraction of a second to run, BFS takes almost the same amount of time as a single objective neural network which is approximately 2 minutes for 1000 epochs. In addition, by integrating the sparse group lasso into the training step of the neural network, the need of an additional level to perform feature selection was eliminated, without adding an extra layer to the network. Moreover, the improvement in FS and sparsification was statistically validated in the case of HDLSS compared to the other methods. By comparing the proposed approach to L1-NN and SGL-NN, the impact of using an appropriate regularization function and multiobjective optimization was demonstrated. Using L1 is not adapted for feature selection in the case of HDLSS data. Furthermore, using an SGL regularizer in a scalarized single objective favors feature selection but not sparse networks. It is worth noting that single objective neural networks that use sparse group lasso do not reduce the weights sufficiently to create sparse networks, as it was shown on both artificial and real datasets. In addition, the normalization step added to deal with objective functions of different scales and the constraints increased the diversity of the solutions. MOO-MTL gave better accuracies in general but less diversity in the solutions. Without normalization, solutions tend to differ only on the objective with the highest gradient, which is sparsity in this case. Although the predictions are highly accurate, they cannot be solely attributed to informative features. This is supported by the low *FS* score observed for artificial datasets and the inferior feature ranking for real datasets. This means that the models’ predictions depend on noise or artifacts present in the data rather than on relevant features. In this work, the impact of having the constraints in both the initialization and training steps was also studied by comparing the proposed approach to BFS init only. It was observed that removing the constraints from the training step resulted in a reduction of the Pareto front coverage, leading to less diverse solutions. For instance, in the case AD2, only one solution reached good performance. A more complete and diverse Pareto front enables the user to select the most appropriate solution based on the application’s requirements.

## Conclusion

In this work, it was shown that sparse group lasso regularization is an efficient way to perform simultaneously feature selection and Deep Learning sparsification. The results obtained from experiments on various datasets show that the presented approach, based on a biobjective gradient descent, outperformed the other methods that use regularization approaches regarding selected relevant features. In addition to choosing the most relevant features, this method also has the advantage of generating sparse neural networks. It is worth noting that single objective neural networks that use sparse group lasso do not reduce the weights sufficiently to create sparse networks, as shown on both artificial and real datasets. Another advantage of this approach is that it provides several diverse non-dominated solutions allowing the user to choose the most suitable one according to the requirements of his application. For example, if he has very limited memory resources, he may prefer a very sparse solution instead of a very accurate one.

In future work, this method will be applied to mutation data as they are known to be of high dimensions and low sample size. In the health field and more specifically for precision medicine, being able to explain the results obtained or in this case, select the mutations on which to base the prediction is crucial. In addition, in this approach the problem of generating a discrete Pareto front that contains well-dispersed solutions was addressed. So far, very few works have addressed the problem of generating continuous Pareto front for multi-task learning and fairness problems based on hypernetworks [43]. These approaches will be studied in the context of feature selection for HDLSS data.

However, BFS has a few limitations. First, sparse group lasso does not take into account redundant and correlated features. This problem can be addressed by using a different objective function rather than the sparse group lasso. In addition, the feature selection step is based on an *FS* score computed directly on the weights. While this method has been used frequently used in deep learning and gives reliable results, there exist other methods that can be more accurate such as LRP [44]. This will be addressed in future work. Finally, as pointed out in [37] solutions will only stay in their regions if the number of regions is large. One way to overcome this, is by using Exact Pareto Optimal search which has proved to be efficient in some multi-task learning in deep learning as proposed by the authors. This idea will be investigated in the case of feature selection.

## Supporting information

S1 Text The source code, datasets, and appendices are available at https://forge.ibisc.univ-evry.fr/tissa/BFS. (DOCX)

S1 Appendix (ZIP)

S2 Appendix (ZIP)
