# Supplementary material for: Biobjective gradient descent for feature selection on high dimension, low sample size data
Source: PLoS One. 2024 Jul 18;19(7):e0305654. doi: 10.1371/journal.pone.0305654 (PMC11257339; doi:10.1371/journal.pone.0305654)
Supplement: S1 Text — (DOCX) [file pone.0305654.s001.docx]

S1 Text. The source code, datasets, and appendices are available at

https://forge.ibisc.univ-evry.fr/tissa/BFS
