# Supplementary material for: Biobjective gradient descent for feature selection on high dimension, low sample size data
Source: PLoS One. 2024 Jul 18;19(7):e0305654. doi: 10.1371/journal.pone.0305654 (PMC11257339; doi:10.1371/journal.pone.0305654)
Supplement: S1 Appendix — (ZIP) [file pone.0305654.s002.zip › Appendix_A.pdf]

Appendix A. Unconstrained multiobjectve gradient descent methods: Definitions and theorems

Appendix A.1. Unconstained multiobjectve gradient descent methods: Definitions

Let $F : \mathbb{R}^n \to \mathbb{R}^m$ be a function with $F(x) = (f_1(x), f_2(x), \ldots, f_m(x))$ such that each $f_i : \mathbb{R}^n \to \mathbb{R}$, $i = 1, \ldots, m$, is a differentiable function. Recall that the gradient of a function f_i at solution $x = (x_1, x_2, \ldots x_n)$, is defined as $\nabla f_i(x) = \left(\frac{\partial f_i}{\partial x_1}(x), \frac{\partial f_i}{\partial x_2}(x), \cdots, \frac{\partial f_i}{\partial x_n}(x)\right)$. In this section, we consider the following unconstrained multiobjective optimization problem:

$$\min_{x \in \mathbb{R}^n} F(x). \tag{A.1.1}$$

One aim of multiobjective optimization is to find the set of non dominated solutions, called the Pareto set [1] defined next.

Definition 1 (**Pareto dominance**)**.** We say a solution x *dominates* a solution y if $\forall i \in \{1, \ldots, m\}$, we have $f_i(x) \le f_i(y)$ and $\exists j \in \{1, \ldots, m\}$ such that $f_j(x) < f_j(y)$. A solution x^* is said to be *Pareto optimal* if it is not dominated by another solution.

Definition 2 (**Pareto set**)**.** The set of non dominated solutions is called the *Pareto set.*

In practice, finding Pareto optimal solutions is difficult. Several works define necessary conditions for Pareto optimality. The most used are Pareto criticality [2] and Pareto stationarity [3].

Several gradient based methods [2, 3] have been proposed to solve the unconstrained multiobjective optimization problems (A.1.1). Fliege and Svaiter [2] proposed a generalization of the single objective steepest descent algorithm traditionally used in neural networks. In their approach, the update rule of the gradient descent is $x \leftarrow x + l\, d_{FS}$, where l is the step length and $d_{FS} \in \mathbb{R}^n$ the direction. d_{FS} is found by solving the following problem:

$$\begin{aligned} &\min_{d \in \mathbb{R}^n, \gamma \in \mathbb{R}} \gamma + \frac{1}{2}||d||^2 \\ &s.t \quad d.\nabla f_i(x) \le \gamma,\ i = 1, \ldots, m. \end{aligned} \tag{A.1.2}$$

They defined the notion of Pareto criticality and proved the following Lemma 1.

Definition 3 (**Pareto criticality**)**.** A solution x^* is said to be a *Pareto critical solution* iff

$$\nexists d \in \mathbb{R}^n \quad s.t \quad \nabla f_i(x^*).d < 0,\ \forall i \in \{1, \dots, m\}.$$

Lemma 1. [2] Let (d^*_{FS}, γ^*) be the solution of problem (A.1.2).

1. If x is Pareto critical, then $d^*_{FS} = \vec{0}$ and $\gamma^* = 0$.
2. If x is not Pareto critical, then $d^*_{FS} \neq \vec{0}$ and $\gamma^* \leq -\frac{1}{2}||d^*_{FS}||^2 < 0$.

Desideri [3] proposed another approach by introducing the following optimization problem:

$$\begin{aligned} \min_{\alpha \in \mathbb{R}^m} \quad & ||\sum_{i=1}^{m} \alpha_i \nabla f_i(x)||^2 \\ s.t \quad & \alpha_i \geq 0,\ i = 1, ..., m \\ \text{and} \quad & \sum_{i=1}^{m} \alpha_i = 1. \end{aligned} \tag{A.1.3}$$

He defined the notion of Pareto stationarity and proved the following Theorem 1.

Definition 4 (**Pareto stationarity**)**.** A solution x^* is said to be a *Pareto stationary solution* iff there exists a convex combination of the gradient vectors at x^* that is equal to $\vec{0}$:

$$\exists \alpha = (\alpha_1, \dots, \alpha_m) \in \mathbb{R}^m \text{ with } \alpha_i \geq 0,\ \forall i \in \{1, \dots, m\}, \sum_{i=1}^{m} \alpha_i = 1, \text{ and } \sum_{i=1}^{m} \alpha_i \nabla f_i(x^*) = \vec{0}.$$

It means that the objective function has value 0 for the optimum solution of problem (A.1.3).

Theorem 1. [3] Let α^*_i, $i = 1, \dots, m$ be the solution of problem (A.1.3) and $d^*_D = -\sum_{i=1}^{m} \alpha^*_i \nabla f_i(x)$.

1. Either $d^*_D = \vec{0}$, i.e. x is Pareto-stationary;
2. or $d^*_D \neq \vec{0}$ and $-d^*_D$ is a descent direction common to all the criteria.

While reported in the literature, the equivalence between Pareto stationarity and Pareto criticality was never formally proven before, and sometime the terms are used

interchangeably [4]. Another observation which was reported in the literature is the strong link between the two problems (A.1.2) and (A.1.3) via the duality theory [5, 6, 2], however no detailed proof was provided. In this Appendix we prove formally the equivalence between the two definitions as stated in Theorem 2 by using the separating hyperplane theorem without using duality theory. We also formally prove the equivalence of the two directions computed by the Desideri approach and the Fliege & Svaiter approach as stated in Theorem 3 (this equivalence was used in [6]) from which the equivalence between Pareto stationarity and Pareto criticality can be also deduced.

Appendix A.2. Pareto stationarity and Pareto criticality equivalence

Separating hyperplane. Let $\mathscr{H} = \{x \in \mathbb{R}^n : a.x = b\}$ be a hyperplane in $\mathbb{R}^n$ with $a \in \mathbb{R}^n$ and $b \in \mathbb{R}$. Given two subsets C_1, C_2 of $\mathbb{R}^n$, we say that $\mathscr{H}$ separates the two sets, if $C_1 \subseteq \mathscr{H}_-$ and $C_2 \subseteq \mathscr{H}_+$, where:

$$\mathscr{H}_- = \{x \in \mathbb{R}^n : a.x \leq b\},\ \mathscr{H}_+ = \{x \in \mathbb{R}^n : a.x \geq b\}.$$

We say that $\mathscr{H}$ stricly separates C_1, C_2 if $C_1 \subseteq \mathscr{H}_{--}$ and $C_2 \subseteq H_{++}$, where

$$\mathscr{H}_{--} = \{x \in \mathbb{R}^n : a.x < b\},\ \mathscr{H}_{++} = \{x \in \mathbb{R}^n : a.x > b\}.$$

Separating hyperplane theorem. [7] Given two convex subsets C_1, C_2 of $\mathbb{R}^n$ with an empty intersection, there exists a hyperplane $\mathscr{H}$ that separates the two sets. Furthermore, if C_1 is closed and bounded and C_2 is closed, then they can be strictly separated by a hyperplane $\mathscr{H}$.

Theorem 2. A solution x is Pareto stationary iff it is Pareto critical.

Proof. A solution x is Pareto stationary if there exists a convex combination of the gradient vectors at x that is equal to zero. We define

$$C_1 = \{\sum_{i=1}^{n} \alpha_i \nabla f_i(x) : \alpha_i \geq 0\ \forall i \in \{1, \ldots, n\} \text{ and } \sum_{i=1}^{n} \alpha_i = 1\}$$

and we consider the vector $z = \vec{0}$ as C_2.

We suppose x is not a Pareto stationary point, hence, $\vec{0} \notin C_1$. C_1 is a closed and bounded convex set, C_2 is a closed convex set and $C_1 \cap C_2 = \emptyset$. Therefore, by the Separating hyperplane theorem, we can strictly separate C_1 and C_2 by a hyperplane $\mathscr{H}$, and there exists $a \in \mathbb{R}^n$ and $b \in \mathbb{R}$ such that:

$$\begin{aligned} &\forall x \in C_1 : a.x < b, \\ &\forall z \in C_2 : a.z > b. \end{aligned} \tag{A.2.1}$$

Furthermore, we know that $a.z = 0$, then we can deduce that $b < 0$ and $a.x < b < 0$, meaning that:

$$\exists a \in \mathbb{R}^n \quad \text{such that} \quad a.x < 0 \quad \forall x \in C_1 \quad \text{and therefore} \quad a.\nabla f_i(x) < 0, \quad \forall i \in \{1, \ldots, m\}. \tag{A.2.2}$$

Therefore, we conclude that x is not a Pareto critical point. We have proven that if a point is not Pareto stationary then it is not Pareto Critical.

Now, let's assume that a solution x is not Pareto critical, then:

$$\exists d \in \mathbb{R}^n \quad \text{such that} \quad d.\nabla f_i(x) < 0, \quad \forall i \in \{1, \ldots, m\}. \tag{A.2.3}$$

Let's consider any convex combination $\sum_{i=1}^{m} \alpha_i \nabla f_i(x)$, with $\alpha_i \geq 0, i \in \{1, \ldots, m\}$ and $\sum_{i=1}^{m} \alpha_i = 1$. From (A.2.3) we get that:

$$d.(\sum_{i=1}^{m} \alpha_i \nabla f_i(x)) < 0. \tag{A.2.4}$$

Therefore, $\sum_{i=1}^{m} \alpha_i \nabla f_i(x) \neq \vec{0}$ and the Pareto stationarity conditions are not satisfied and x is not a Pareto stationary solution. We have proved that if a solution is not Pareto critical then it is not Pareto stationary as well. ■

Appendix A.3. Equivalence of the two directions computed by the Fliege & Svaiter approach and the Desideri approach

Let us first recall some classical theorems and well known facts about convex optimization (see for example [8]): Let

$$(P)\begin{cases} \min f(x) \\ g_i(x) \leq 0 \quad i = 1, \ldots, m \\ x \in C \subset \mathbb{R}^n \end{cases}$$

be a convex problem for which the Slater's condition is satisfied, i.e there exists a feasible point $x \in C$ such that $g_i(x) < 0$ for $i = 1, \ldots, m$, and let $L(x, \lambda) = f(x) + \sum_{i=1}^{m} \lambda_i g_i(x)$ the Lagrangian of (P), with $\lambda_i \geq 0$ for $i = 1, \ldots, m$, the Lagrange multipliers.

KKT theorem (saddle point form):

$x^* \in C$ is a solution of (P) if and only if $\exists \lambda^* \in \mathbb{R}^m$ such that:

- $\lambda_i^* \geq 0 \;\; i = 1, \ldots, m$;
- $L(x^*, \lambda) \leq L(x^*, \lambda^*) \leq L(x, \lambda^*) \;\; \forall x \in C$ and $\lambda_i \geq 0 \;\; i = 1, \ldots, m$;
- $\lambda_i^* g_i(x^*) = 0 \quad i = 1, \ldots, m$.

KKT theorem (Gradient form):

Let assume that f and g_i have continuous first partial derivatives on C. If x^* is feasible for (P) and an interior point of C, then x^* is a solution if (P) if and only if $\exists \lambda^* \in \mathbb{R}^m$ such that:

- $\lambda_i^* \geq 0 \;\; i = 1, \ldots, m$;
- $\lambda_i^* g_i(x^*) = 0 \;\; i = 1, \ldots, m$;
- $\nabla f(x^*) + \sum_{i=1}^{m} \lambda_i \nabla g_i(x^*) = \vec{0}$.

Remark 1: The dual program of (P) is $\sup_{\lambda \geq 0} \inf_{x \in C} L(x, \lambda)$ and a well known consequence of the KKT theorem (saddle point form) is that

$$\sup_{\lambda \geq 0} \inf_{x \in C} L(x, \lambda) = L(x^*, \lambda^*) = f(x^*). \tag{A.3.1}$$

Now let recall the convex quadratic optimization problem (P_{FS}) used in the Fliege & Svaiter approach ([2]):

$$(P_{FS})\begin{cases} \min\limits_{d\in\mathbb{R}^n,\gamma\in\mathbb{R}} \gamma+\frac{1}{2}||d||^2 \\ \nabla f_i(x).d \le \gamma \quad i=1,\dots,m \end{cases} \tag{A.3.2}$$

Let $(d^*_{FS}, \gamma^*_{FS})$ be the optimal solution. The direction calculated at solution x is d^*_{FS}.

Let recall the quadratic optimization problem (P_D) used in the Desideri approach ([3]):

$$(P_D)\begin{cases} \min\ ||\sum_{i=1}^m \alpha_i \nabla f_i(x)||^2 \\ \sum_{i=1}^m \alpha_i = 1 \\ \alpha_i \ge 0,\ i=1,\dots,m \end{cases}$$

Let α_i^* be the optimal solution and $d^*_D = -\sum_{i=1}^m \alpha_i^* \nabla f_i(x)$ the direction calculated at solution x. We have the following theorem.

Theorem 3. Let d^*_{FS} (resp. d^*_D) be the direction computed by the Fliege & Svaiter (resp. Desideri) approach. One has $d^*_{FS} = d^*_D$.

Proof. Let us observe that (P_{FS}) is a convex problem for which the Slater's condition holds, i.e.$\exists \gamma\in\mathbb{R}, d\in\mathbb{R}^n$ such that $\nabla f_i(x).d < \gamma$ for $i=1,\dots,m$.

From the KKT theorem (Gradient form) it is easy to get that (see [9]) if $\lambda_1^*,\dots,\lambda_m^* \ge 0$ are the optimal Lagrange multipliers of the problem (P_{FS}) then one has:

$$\begin{aligned} d^*_{FS} &= -\sum_{i=1}^m \lambda_i^* \nabla f_i(x), \\ \gamma^*_{FS} &= -||d^*_{FS}||^2, \\ \sum_{i=1}^m \lambda_i^* &= 1. \end{aligned} \tag{A.3.3}$$

The Lagrangian of (P_{FS}) is

$$L(\gamma,d,\lambda) = \gamma + \frac{1}{2}||d||^2 + \sum_{i=1}^m \lambda_i(\nabla f_i(x).d - \gamma),$$

and one gets for the dual program of (P_{FS})

$$\begin{aligned}\max_{\lambda\geq 0}\inf_{\gamma\in\mathbb{R},d\in\mathbb{R}^n} L(\gamma,d,\lambda) &= L(\gamma^*_{FS},d^*_{FS},\lambda^*)\\ &= \gamma^*_{FS}+\frac{1}{2}||d^*_{FS}||^2+\sum_{i=1}^{m}\lambda^*_i(\nabla f_i(x).d-\gamma^*_{FS})\\ &= -\frac{1}{2}\sum_{i=1}^{m}||\lambda^*_i\nabla f_i(x)||^2 \qquad \text{(using (A.3.3))}\end{aligned}$$

where the only constraints on λ^*_i are $\sum_{i=1}^m \lambda^*_i = 1$ and $\lambda^*_i \geq 0$ for $i = 1,\dots,m$. Therefore, the dual program of (P_{FS}) can be written as

$$dual(P_{FS})\begin{cases}\min\ \frac{1}{2}||\sum_{i=1}^m \lambda_i\nabla f_i(x)||^2\\ \sum_{i=1}^m \lambda_i = 1\\ \lambda_i \geq 0,\ i=1,\dots,m\end{cases} \tag{A.3.4}$$

which is the same as (P_D) excepted that the objective function is divided by 2. Therefore when we solve the (P_D) problem, we get $\alpha^*_i = \lambda^*_i$ for $i = 1,\dots,m$, and $d^*_{FS} = -\sum_{i=1}^m \lambda^*_i\nabla f_i(x) = -\sum_{i=1}^m \alpha^*_i\nabla f_i(x) = d^*_D$. ■

Remark 2: Notice that Theorem 3 can be viewed as a corollary of Theorem 2 since a solution is Pareto stationary (resp. Pareto critical) iff $d^*_D = \vec{0}$ (resp. $d^*_{FS} = \vec{0}$).

Remark 3: Notice that in problem (5) the constraints $\nabla f_i(\theta).d \leq \gamma$, $i = 1,\dots m$ have the same "shape" than the constraints $\nabla G^k_j(\theta).d \leq \gamma$, $j \in I_\epsilon(\theta)$, therefore given that the problem (A.3.4) is the dual of the problem (A.3.2) it is straightforward to derive that the problem (6) is the dual of the problem (5).

References

[1] M. Ehrgott, Multicriteria Optimization, Springer, 2005.

[2] J. Fliege, B. F. Svaiter, Steepest descent methods for multicriteria optimization, Mathematical methods of operations research 51 (3) (2000) 479–494.

[3] J.-A. Désidéri, Multiple-gradient descent algorithm (MGDA) for multiobjective optimization, Comptes Rendus Mathematique, Académie des Sciences (Paris) 350 (5-6) (2012) 313–318.

[4] G. Cocchi, G. Liuzzi, S. Lucidi, M. Sciandrone, On the convergence of steepest descent methods for multiobjective optimization, Computational Optimization and Applications 77 (2020) 1–27.

[5] S. Liu, L. N. Vicente, The stochastic multi-gradient algorithm for multi-objective optimization and its application to supervised machine learning, Annals of Operations Research (2021) 1–30.

[6] X. Lin, H.-L. Zhen, Z. Li, Q.-F. Zhang, S. Kwong, Pareto multi-task learning, in: Advances in Neural Information Processing Systems – NIPS'19, 2019, pp. 12060–12070.

[7] G. C. Calafiore, L. El Ghaoui, Optimization models, Cambridge university press, 2014.

[8] A. Peressini, F. Sullivan, J. Uhl, The Mathematics of nonlinear programming, Springer, 1998.

[9] L. Zeng, Y. Dai, Convergence rate of gradient descent method for multi-objective optimization, Journal of Computational Mathematics 37 (5) (2019) 689–703.
