# Supplementary material for: Biobjective gradient descent for feature selection on high dimension, low sample size data
Source: PLoS One. 2024 Jul 18;19(7):e0305654. doi: 10.1371/journal.pone.0305654 (PMC11257339; doi:10.1371/journal.pone.0305654)
Supplement: S2 Appendix — (ZIP) [file pone.0305654.s003.zip › AppendixB.pdf]

Appendix B

1 Implementation details

1.1 Artificial datasets

We generate two artificial datasets in order to evaluate our method. We use the *make_classification* function from scikit-learn [1]. For AD1, we set the number of total features to 2000 with 50 informative features, 0 redundant, 0 repeated and 1950 useless features drawn at random. The dataset consists of two classes, each made up of one cluster. For AD2, we set the number of total features to 3000 with 50 informative features, 0 redundant, 0 repeated, and 2950 useless features drawn at random. We have three classes, each one consisting of two clusters. The fraction of samples whose class is assigned randomly for both datasets is 0.01. We provide both datasets and the code to generate artificial datasets at `https://forge.ibisc.univ-evry.fr/tissa/BFS`.

1.2 Hyperparameter search for each method

Methods are all developed and implemented using Python 3 and Tensorflow 2 [2], and all experiments were run on Linux with GPUs of type RTX 2080Ti, 3080Ti, and 3090. The dual problem in BFS was solved using the cvxopt solver [3]. For MOO-MTL, we solved the problem with the method proposed in the code provided by the authors.

Each dataset is split into train and test sets. The weights of the neural networks are initialized by drawing from $\mathcal{N}(0, 0.05)$, and the bias was set to 0. We use Random Search and cross-validation (5-fold CV) to tune the hyperparameters. The list of hyperparameters used for all methods and the search range are summarized in table 1.

Parameter	Search range
# of dense layers	$[1, 6]$
# of hidden units	$[10, 500]$
Learning rate	$[10^{-4}, 10^{-1}]$
λ (SGL)	$[0, 1]$
# of epochs	$[150, 1000]$
batch size	$[8, 256]$
optimizer	SGD, Adam, Momentum, Adagrad, Adadelta

Table 1: List of hyperparameters used as well as their search range.

After finding the best hyperparameters using random search and 5-fold CV, we run each method on each dataset 100 times so we always end up with the same number of final solutions. For BFS and BFS init, the initial objective space is split into 100 regions to generate 100 solutions. We use the ReLU activation function for all experiments for the hidden layer and the softmax activation function for the output layer.

For the AD1 dataset, for L1-NN, the architecture that gave the best results was 2 hidden layers with [128, 416] neurons, respectively. The best optimizer was Adagrad, with a learning rate of 0.74, 664 epochs, and a batch size of 49. For SGL-NN, the best architecture was 3 hidden layers with [379, 89, 296] neurons respectively. The best optimizer was Adam, with a learning rate of 0.6, and the regularization term for the sparse group lasso was 0.26. The number of epochs is 491, and the batch size is 49. For MOO-MTL, the best optimizer was Adam with a learning rate of 0.3 and 401 epochs and batches of size 22. The best architecture was made up of 4 hidden layers with

[1] https://scikit-learn.org/stable/
[2] https://www.tensorflow.org
[3] https://cvxopt.org

[316, 10, 327, 457] neurons respectively. We used the same parameters for BFS and BFS init only, as they gave the best results. The best optimizer was Adam, with a learning rate of 0.01 for both the initialization and training optimizers. The best architecture consisted of 3 hidden layers with [186,286,53] neurons respectively. Finally, contrary to the other methods, the optimal batch size was high, with a value of 128.

For the AD2 dataset, for L1-NN, the architecture with that gave the best results was 4 hidden layers with [399, 482, 233, 320] neurons respectively. The best optimizer was SGD with a learning rate of 0.5 and with 237 epochs and a batch size of 51. For SGL-NN, the best architecture was 4 hidden layers with [33,60 202, 19] neurons respectively. The best optimizer was Adagrad, with a learning rate of 0.4, and the regularization term for the sparse group lasso was 0.73. The number of epochs is 334, and the batch size is 55. For MOO-MTL, the best optimizer was Adagrad with a learning rate of 0.4 and with 412 epochs and batches of size 29. The best architecture was made up of 6 hidden layers with [395, 46, 342, 292, 490, 461] neurons respectively. Except for the architecture, we used the same hyperparameters for BFS and BFS init only. The best optimizer was Adam, with a learning rate of 0.01 for both the initialization and training optimizers. For BFS, the best architecture consisted of 3 hidden layers with [100,50,14] neurons, respectively. For BFS init only, the best architecture consisted of 3 layers with [206, 170, 23] respectively. Finally, contrary to the other methods, the optimal batch size was high, with a value of 129.

For the BASEHOCK dataset, for L1-NN, the architecture that gave the best results was 3 hidden layers with [401, 382, 344] neurons, respectively. The best optimizer was Adam with a learning rate of 0.3 and with 974 epochs and a batch size of 23. For SGL-NN, the best architecture was 2 hidden layers with [478, 460] neurons, respectively. The best optimizer was Momentum with a learning rate of 0.7, a momentum rate of 0.8 and the regularization term for the sparse group lasso was 0.9. The number of epochs is 610, and the batch size is 23. For MOO-MTL, the best optimizer was Adagrad with a learning rate of 0.4 and with 517 epochs and batches of size 34. The best architecture was made up of 5 hidden layers with [488, 301, 221, 298, 284] neurons respectively. Except for the architecture, we used the same hyperparameters for BFS and BFS init only. The best optimizer was Adam with a learning rate of 0.03 for both the initialization and training optimizers. For BFS, the best architecture consisted of 2 hidden layers with [100,50] neurons respectively. For BFS init only, the best architecture consisted of 3 layers with [193, 46, 25] respectively. Finally, contrary to the other methods, the optimal batch size was high, with a value of 130.

For the PCMAC dataset, for L1-NN, the architecture that gave the best results was 4 hidden layers with [114, 129, 91, 215] neurons, respectively. The best optimizer was Adam with a learning rate of 0.4 and with 474 epochs and a batch size of 10. For SGL-NN, the best architecture was 3 hidden layers with [201, 238, 258] neurons respectively. The best optimizer was SGD with a learning rate of 0.9, and the regularization term for the sparse group lasso was 0.8. The number of epochs is 360, and the batch size is 23. For MOO-MTL, the best optimizer was Adam, with a learning rate of 0.3, 300 epochs, and batches of size 50. The best architecture was made up of 5 hidden layers with [72, 423, 445, 29, 24] neurons respectively. Except for the architecture, we used the same hyperparameters for BFS and BFS init only. We used the same parameters for BFS and BFS init only, as they gave the best results. The best optimizer was Adam with a learning rate of 0.02 for both the initialization and training optimizers. The best architecture consisted of 3 hidden layers with [108, 59, 23] neurons respectively. Finally, contrary to the other methods, the optimal batch size was high, with a value of 127.

1.3 Comparison to ParetoMTL

We tried comparing our method to ParetoMTL. For this, we first tested both ParetoMTL and BFS on a synthetic example we define later in Section 2.1. No GPU acceleration was used when dealing with the synthetic example. We start by testing with 10 regions. When the two objective functions are imbalanced, it takes approximately 26 seconds for BFS to run and almost 60 seconds for ParetoMTL to finish running. We then increased the number of regions to 100, using the same imbalanced objective functions. It takes 397 seconds for BFS to finish running, whereas it takes 1931 seconds for ParetoMTL to finish running. The number of regions drastically increases the running time of ParetoMTL, which is expected as the dual problem is directly dependent on the

number of regions. It is worth noting that there were only 20 input variables in the synthetic example.

However, we then wanted to test ParetoMTL on neural networks. We used a Linux environment with GPU GeoForce RTX 2080Ti acceleration. ParetoMTL was written in Pytorch and was used only on Convolutional Neural Networks (CNN). We rewrite a version that can be used with MLP and TensorFlow. With BFS, regardless of the number of regions, a network took approximately 2 minutes for a network to train. Unfortunately, we could not test it on ParetoMTL as the algorithm was only at the second neural network after 3 days and with only 10 regions. This means that to obtain our 100 regions, we would have had to wait at least 150 days.

2 Supplementary results

2.1 Synthetic example

Before applying our method to neural networks, we started by verifying that our method does indeed satisfy two goals of multiobjective optimization. The first is to converge to the Pareto front, and the second is to generate dispersed solutions. Since it is difficult to assess both goals with the same performance metric, we will use, in addition to the Pareto front visualization, two metrics, the convergence metric, and the spread score, to evaluate the different methods. The metrics were defined in [1] We note that these methods apply if the real Pareto front is known.

- The convergence metric, Υ, is used to evaluate if the optimal solutions obtained by an algorithm are on the real Pareto front or not. To obtain this score, we need to know, a priori, the set of Pareto optimal solutions and select H a subset of these solutions. Let $X = \{x_1, x_2, \ldots, x_n\}$ be the set of non-dominated solutions obtained by an algorithm, we define Υ as:
$$\Upsilon = \frac{1}{n}\sum_{i=1}^{n} \min_{h \in H} d(x_i, h) \tag{1}$$
where $d(x_i, h)$ represents the Euclidean distance between x_i and h. A score of 0 indicates that all obtained solutions are among the chosen solutions H. The smaller Υ is, the better the convergence towards the Pareto front. A small score close to 0 signifies that the non-dominated solutions are possibly on the Pareto front but not among the H selected Pareto optimal set.

- The spread score, Δ, is used to evaluate the diversity of the obtained non-dominated solutions in terms of spread and uniformity, regardless of whether they are on the real Pareto front or not. To calculate this score, we need to know *a priori* the real extreme points of the Pareto front. Δ is defined as:
$$\Delta = \frac{d_u + d_l + \sum_{i=1}^{n-1} |d_i - \overline{d}|}{d_u + d_l + (n-1)\overline{d}} \tag{2}$$
where d_u and d_l are the Euclidean distances between the extreme solutions and the boundary solutions of the obtained non-dominated set, and d_i is the distance between two consecutive solutions. Let n be the number of solutions obtained; then there would be $(n-1)$ consecutive distances. $\overline{d}$ is the average of all d_i distances. When the extreme points are in the non-dominated set ($d_u = d_l = 0$) and all solutions are uniformly spread out, Δ takes a value of 0. Hence, the lower the value of Δ, the more diverse the Pareto front.

We choose the synthetic multi-objective example introduced in [2] to test our method, and we compare the obtained results to those obtained by the authors (ParetoMTL) as well as other state-of-the-art methods, mainly a linear method and MOO-MTL [3]. The general formulation of the problem is defined below:

$$\min_x f_1(x) = \alpha_1 - \alpha_1 \times \exp\left(-\sum_{i=1}^{d}(x_d - \frac{1}{\sqrt{d}})^2\right)$$
$$\min_x f_2(x) = \alpha_2 - \alpha_2 \times \exp\left(-\sum_{i=1}^{d}(x_d + \frac{1}{\sqrt{d}})^2\right) \quad (3)$$

In the above problem, depending on the values α_1 and α_2 take, the problem's difficulty varies. In fact, when $\alpha_1 = \alpha_2$, the two objective functions are balanced, and it is the easiest variant to solve. However, as the difference between the values of α_1 and α_2 increases, the two objective functions become unbalanced, and harder it is to solve the problem. For instance, if $\alpha_1 > \alpha_2$, then f_1 can be easier to solve than f_2 due to the larger value of its gradient.
In our case, we set $\alpha_2 = 1$ and we vary α_1 to create four different scenarios: balanced ($\alpha_1 = 1$), easy unbalanced ($\alpha_1 = 2$), intermediate unbalanced ($\alpha_1 = 10$) and hard unbalanced ($\alpha_1 = 50$).
Figure 1 shows the obtained Pareto fronts for each scenario (Fig(1A-D)) as well as the running time for each of the compared methods(Fig(1E)). Visually, we can see that in the case of balanced, objective functions, all methods give optimal solutions that are on the real Pareto front. However, only BFS and ParetoMTL give dispersed solutions. The linear method is only able to detect the extreme points. For all four scenarios, BFS is the only method to reach at least one of the extreme points as well as diverse solutions on the Pareto front. We also notice that the more unbalanced the objective functions are, the harder it is for ParetoMTL and the linear method to reach the real Pareto front. Lastly, we can see that while MOO-MTL manages to find solutions from the optimal set, they are all gathered on one area of the Pareto front. The more unbalanced the two functions, the closer the found solutions are to the objective function with the bigger values. Regarding running time, we see that while all methods have a similar running time when the objective functions are balanced, ParetoMTL's running time increases as the problem becomes harder to solve. This running time is double the time our method requires when the problem is hard. The formulation of BFS, with at most one constraint, makes it competitive with traditional methods. While visualizing the Pareto fronts gives us an idea of how well each method performed, we support our findings by calculating the convergence metric and the spread score for each method and each scenario. We summarize the obtained results in Table (2) below.

Method	Balanced		Easy		Intermediate		Hard	
	Convergence	Spread	Convergence	Spread	Convergence	Spread	Convergence	Spread
BFS	4.3×10^{-5}	**0.25**	6.4×10^{-5}	**0.33**	$\mathbf{4.4 \times 10^{-5}}$	**0.42**	2.8×10^{-4}	**0.54**
ParetoMTL	1.1×10^{-4}	0.77	1.2×10^{-4}	0.85	8.8×10^{-4}	0.94	9.9×10^{-3}	0.86
MOO-MTL	3.4×10^{-5}	0.86	3.0×10^{-4}	0.87	1.6×10^{-4}	0.84	$\mathbf{9.1 \times 10^{-5}}$	0.92
Linear	$\mathbf{2.9 \times 10^{-6}}$	0.82	$\mathbf{2.0 \times 10^{-5}}$	1.08	1.6×10^{-4}	1.00	0.15	-

Table 2: Summary of convergence metric and spread score for all methods and all scenarios.

From the results we summarized in Table (2), we can deduce that BFS had the most diverse solutions in all four scenarios, as it had the lowest spread score. Like what the obtained Pareto fronts show, the low convergence score, very close to 0, reconfirms that all methods have solutions on the real Pareto front except for the linear method in the case of highly unbalanced objective functions. Overall, we can say that BFS has the best results on all four tested problems in terms of convergence, diversity of solutions, and running time.

References

1. Deb K, Pratap A, Agarwal S, Meyarivan T. A fast and elitist multiobjective genetic algorithm: NSGA-II IEEE transactions on evolutionary computation;2002;6(2):182–197

2. Lin X, Zhen HL, Li Z, Zhang QF, Kwong S. Pareto multi-task learning. In: Advances in Neural Information Processing Systems – NIPS'19; 2019. p. 12060–12070.

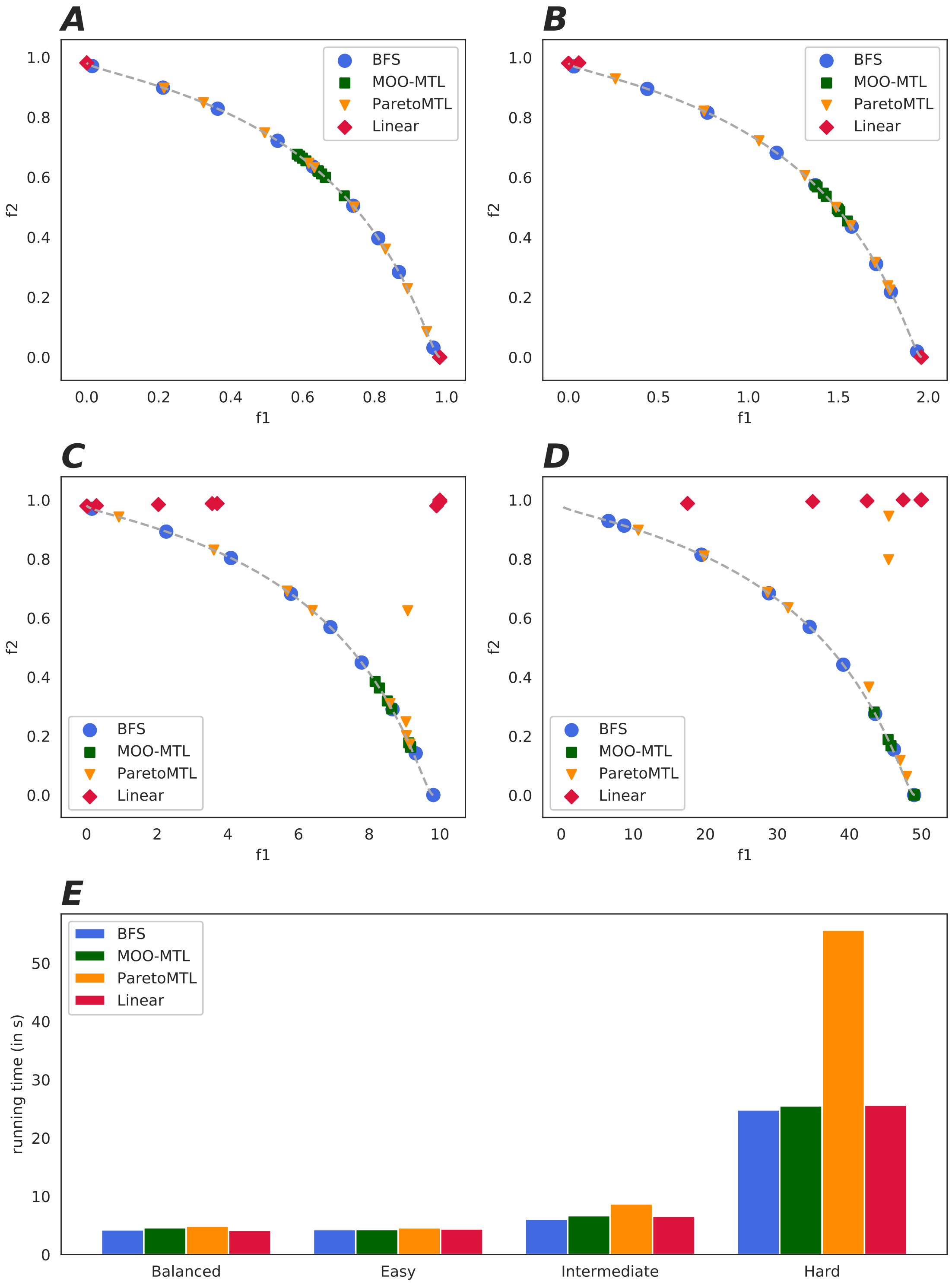

Fig 1: Results summary on synthetic problem with increasing difficulty. Figure showing convergence and running time obtained on problems with different difficulties (balanced, easy, intermediate, and hard) using four different algorithms: BFS (our method), MOO-MTL, ParetoMTL, and linear scalarized method. (A) shows the results obtained when the two tasks are balanced ($\alpha_1 = \alpha_2 = 1$). (B) shows the results obtained when the problem is an easy unbalanced problem($\alpha_1 = 2, \alpha_2 = 1$). The results obtained when the unbalanced problem is of intermediate difficulty ($\alpha_1 = 10, \alpha_2 = 1$) are shown in (C), whereas the results of the hard unbalanced problem ($\alpha_1 = 50, \alpha_2 = 1$) are displayed in (D). The dashed line in all four figures represents the real Pareto set. The running time in seconds for each problem and each method are shown in (E).

3. Sener O, Koltun V. Multi-Task Learning as Multi-Objective Optimization. In: Proceedings of the 32nd International Conference on Neural Information Processing Systems – NIPS'18. NIPS'18. Curran Associates Inc.; 2018. p. 525–536.
